# Modelling the performance of an integrated fixed-film activated sludge (IFAS) system: a systematic approach to automated calibration

**DOI:** 10.1038/s41598-022-13779-w

**Published:** 2022-06-08

**Authors:** D. Pryce, Z. Kapelan, F. A. Memon

**Affiliations:** 1 College of Engineering, Mathematics and Physical Science, University of Exeter, Exeter, UK; 2 Faculty of Civil Engineering and Geosciences, Delft University of Technology, Delft, The Netherlands

**Keywords:** Environmental biotechnology, Civil engineering, Computational models

## Abstract

IFAS systems are inherently complex due to the hybrid use of both suspended and attached bacterial colonies for the purpose of pollutant degradation as part of wastewater treatment. This poses challenges when attempting to represent these systems mathematically due to the vast number of parameters involved. Besides becoming convoluted, large effort will be incurred during model calibration. This paper demonstrates a systematic approach to calibration of an IFAS process model that incorporates two sensitivity analyses to identify influential parameters and detect collinearity from a subset of 68 kinetic and stoichiometric parameters, and the use of the Nelder–Mead optimization algorithm to estimate the required values of these parameters. The model considers the removal of three critical pollutants including biochemical oxygen demand (BOD), total nitrogen (TN) and total suspended solids (TSS). Results from the sensitivity analyses identified four parameters that were the primary influence on the model. The model was found to be most sensitive to the two stoichiometric parameters including aerobic heterotrophic yield on soluble substrate whose total effects were responsible for 92.4% of the model’s BOD output sensitivity and 92.8% of the model’s TSS output sensitivity. The anoxic heterotrophic yield on soluble substrate was observed to be responsible for 54.3% of the model’s TN output sensitivity. To a lesser extent the two kinetic parameters, aerobic heterotrophic decay rate and reduction factor for denitrification on nitrite, were responsible for only 8.0% and 13.1% of the model’s BOD and TN output sensitivities respectively. Parameter estimation identified the need for only minor adjustments to default values in order to achieve sufficient accuracy of simulation with deviation from observed data to be only ± 3.6 mg/L, ± 1.3 mg/L, and ± 9.5 mg/L for BOD, TN and TSS respectively. Validation showed the model was limited in its capacity to predict system behaviour under extreme dissolved oxygen stress.

## Introduction

Computational process models of wastewater treatment plants (WWTP) have long been utilized for the benefits they afford^1^. Such benefits may include the investigation of alternative design and operational scenarios in pursuit of improved efficiency or performance, or to gain insight into system behaviour under circumstantial scenarios^2^. Through modelling, the high demand of such investigations on time and resources that would be accrued with physical experimentation can be overcome with relative ease without compromising on confidence in the results. For example, a model can allow a process engineer to identify the size of anoxic tank required to enable sufficient denitrification in a system without building many different sized anoxic tanks.

While complex models can provide insights to the simulated system that would otherwise be obscured by simplified models, this only holds true if the physical system is accurately depicted. The calibration of biological WWTP models is typically the most demanding phase of model development in terms of time, effort and financial resources required to collect necessary data^2–5^. In fact several protocols such as BIOMATH, STOWA, WERF and HSG have been developed to guide the calibration of activated sludge (AS) models^6–10^. While each of these approaches offer distinct differences, they share common demands for large amounts of data that must be yielded by way of site survey, intensive sampling and respirometric/titrimetric batch tests that place high demands on resources (see ^3^ for a critical review).

As environmental models, biological wastewater treatment (WWT) process models are inherently complex. By their very nature, they are highly-dimensional and non-linear due to the vast array of kinetics and stoichiometry that are included. For instance, even the popular Activated Sludge Model (ASM) in its earliest form consisted of five stoichiometric parameters to describe the biochemical reactions, 14 kinetic parameters and 13 differential equation^1^. It is no surprise then that WWT models become over-parameterised with regards to given observation when these models are further developed to provide a more detailed representation of the underlying processes or to model emerging technologies and contaminants^11–14^.

Increased complexity is unavoidable when multiple technologies are combined such as the more recent integrated fixed-film activated sludge (IFAS) solution. This technology combines the application of suspended biomass colonies with attached biofilm colonies to draw on the advantages of each, while increasing functional biomass and therefore treatment capacity at a reduced plant footprint compared to conventional AS or biofiltration systems^15–18^. The main limitation of this technology however is the substantial increase in energy demand for aeration compared to AS^19^, which necessitates continued modelling efforts towards optimizing its efficiency.

While IFAS has been the subject of much modelling work to date ^15,20–23^, the calibration procedure has received little attention in contrast to AS models despite the added intricacy. Brockmann et al. ^24^ attempted to calibrate an IFAS system by following the good biofilm reactor modelling practice (GBRMP) proposed by Boltz et al.^25^, however the authors found this was inadequate for this purpose, proposing the need for further development of a specific protocol for this technology. A major challenge the authors highlighted was the over-parameterization associated with the hybridization of the two components.

Automatic calibration has received increasing interest over the last few decades ^10,26–29^. While manual calibration by way of experimentally-determining parameter value estimates is conventional, this process remains laborious and resource-heavy and may introduce errors while lacking objectivity^3,30^. Furthermore it is ineffective in estimating the many unmeasurable parameters found in WWT models ^31^. Automatic calibration refers to the use of an optimization algorithm to estimate the parameter values and there are increasing examples of this approach being taken in the modelling of wastewater treatment and waste collection systems^26,30,32–35^.

Kim et al. ^32^ demonstrated the feasibility of using a genetic algorithm (GA) to calibrate the first ASM (ASM1), while a study by Zeferino et al. ^26^ into the model planning of a regional wastewater system utilized a Particle Swarm Optimization (PSO) algorithm to calibrate their model. Ye ^34^ also reported the use of an immune algorithm (IA) and a hybridized form of the IA and PSO (IPSO) to calibrate an ASM. In developing the numerical optimal approaching procedure (NOAP) for systematically calibrating the third of the ASM series (ASM3), Zhu et al. ^10^ employed a genetic algorithm (GA) to successfully automate the parameter estimation of two AS system types. More recently Du et al. ^35^ employed an improved cuckoo search (ICS) algorithm to calibrate up to 7 sensitive parameters in ASM1 yielding accurate simulation.

In the current work a classical optimization technique is employed, the Nelder–Mead (NM) simplex method^36^. While the last few decades have seen an accelerating growth of novel optimization algorithms (see ^37^ for review), the NM simplex approach remains relevant because it is robust, easy to implement and understand and is a favoured algorithm for dealing with multi-dimensional, unconstrained optimization problems without derivatives^38^. Despite being a vintage approach to optimization it has recently been shown to remain competitive in performance with more “intelligent” algorithms such as the PSO algorithm^39^.

As a proceeding step to the parameter estimation, global sensitivity analyses (GSAs) were employed to deal with the issue of over-parameterisation in the IFAS model and reduce the optimization problem to a manageable number of parameters. Regardless of whether a manual or automatic calibration approach is being taken, GSAs can mitigate over-parameterisation by identifying the parameters most influential to the model output that warrant the most focus and resource allocation while leaving less influential parameters at default values^9,10,40–45^. This was a primary concern of Gernaey et al.^2^ who argued that the automated calibration approach may be stifled by a lack of identifiability in parameters which may lead to only minor adjustments in considerable number of secondary parameters.

The use of GSA during calibration afford additional benefits to the process. While they can identify the most critical parameters for estimation, they are also able to check for collinearity of parameters by investigating higher order interactions^43,45,46^. These are both important because non-influence and collinearity with other parameters are the two main sources of practical parameter non-identifiability^13^. Furthermore, because GSA allows the modeller to apportion the uncertainty that is propagated through the model due to each parameter and their interactions^45^, this may be coupled with a Monte-Carlo based uncertainty analysis to then quantify the uncertainty for each factor installing confidence in the model^47–49^.

The objectives of this study are therefore to identify the kinetic and stoichiometric parameters most influential to the model, to estimate the values of any identified parameters by way of an optimization algorithm and to validate the NM simplex algorithm as remaining an appropriate and effective tool for this role. Finally, uncertainty of the model outputs relating to the identified influential parameters are also assessed by way of uncertainty analysis.

## Methodology

### Data collection

Due to travel limitations relating to the COVID-19 pandemic, access to the modelled system was prevented. Under these circumstances historic data was instead required for the purpose of calibration and validation. Fortunately a pilot-scale version of the modelled WWT system had been operated in Rishikesh, Uttar Pradesh, India over the course of 2015 as a research platform for this new technology. During this time it was subject to intense investigation that resulted in a series of publications^50–55^. Most notably was a study into the effects of DO stress on the system^51^. In this study a detailed account of influent and effluent data was provided alongside the operational strategy of three DO concentrations (0.5, 2.5, 4.5 mg/L). For the purpose of the present work, data from the 2.5 mg/L DO regime was used as observed data for the calibration, while the remaining two regimes were utilized in the validation phase as independent data. Investigation from the calibration regime was reported in greater detail in an earlier publication^50^.

### Subject system to be modelled

The system itself consisted of an aerated reactor (20 m^3^) with a 6 m^2^ footprint and a separate circular settling tank with sloping bottom totalling a volume of 4.2 m^3^ and a surface loading area of 1.25 m^2^. The aeration tank contained 64 rectangular panels of loop knitted polypropylene fabric (Biotextile Cleartec^®^, Jager, Germany) that occupied ~ 0.5% of the reactor volume in order to facilitate the proliferation of attached biomass. Further details regarding dimensions are provided in other works^51,55^.

Site conditions for the model reflected the conditions of DO regime 2 (i.e. 2.5 mg/L) as reported by Singh et al. ^51^ for model calibration. These include a flow rate (Q) of 1.8 m^3^/h to give a hydraulic retention time (HRT) of 11.1 h, bulk liquid temperature of 26 °C, DO concentration within the bulk liquid of the HySAF reactor of 2.5 mg/L for model calibration. The activated sludge waste (WAS) rate was set to 2.2 m^3^/day with a maintained recycle activated sludge (RAS) rate of 3.7 m^3^/h (2.5 Q) to ensure a mixed liqour suspended solids (MLSS) concentration of 2000 mg/L in the reactor and a sludge retention time (SRT) of 11 days as reported by Singh et al.^51^. Operational characteristics of DO regimes 1 and 3 as shown in Singh et al. ^51^ were used for validation purposes following the calibration.

### Model development

The commercial modelling software GPS-X™ Version 8.0 (Hydromantis Environmental Software Solutions, Inc.) was used to model the system. This software, while robust and feature-rich, was chosen particularly for its recent integration of the Python coding language in the latest installment that facilitated the use of external Python libraries to perform subsequent analyses in this study.

**Figure 1:**
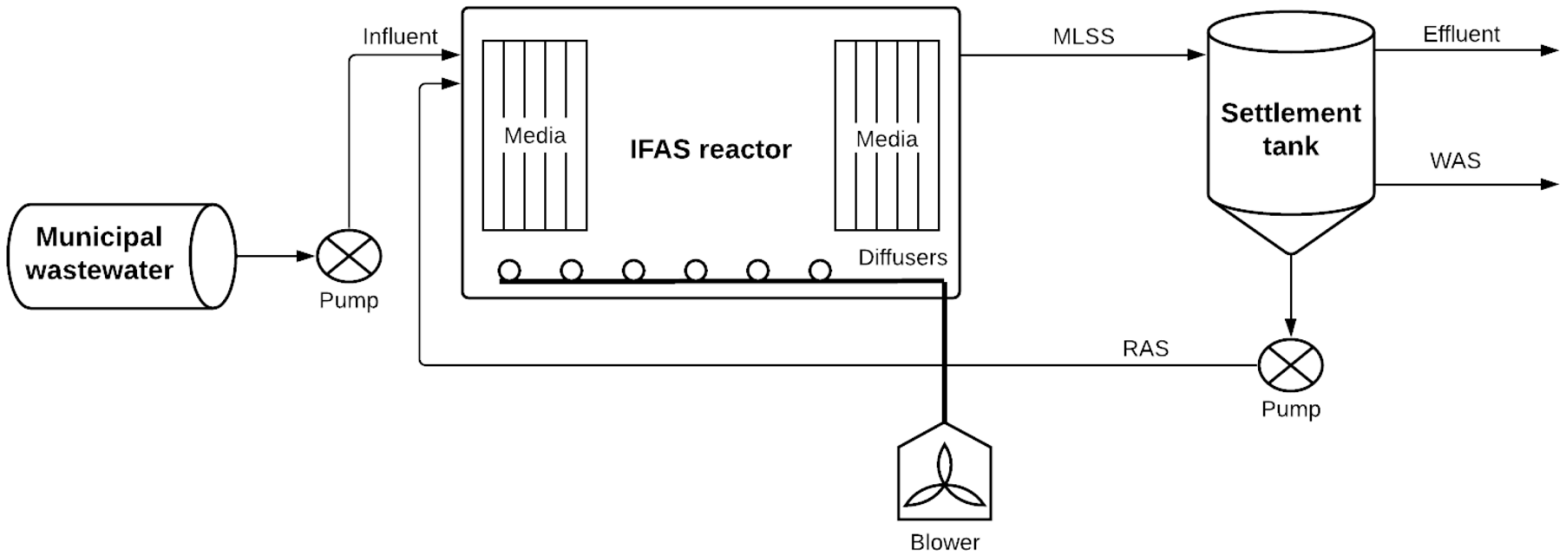
Modelled system diagram.

As shown in Fig. 1, the developed model comprised of the following objects:

- wastewater (WW) influent from the sewer,
- aerobic IFAS reactor,
- settlement tank (secondary).

Figure 1 also displays several key parameters that needed to be defined within the model objects including the mixed liquor suspended solids (MLSS) concentration, the recycle activated sludge (RAS) stream, the waste activated sludge (WAS) stream and media content within the IFAS reactor.

### Wastewater influent from the sewer

The influent model is commonly regarded as the most important element during model calibration^3^. For the purpose of this study the “CODSTATES” model was chosen from the Comprehensive Model Library (MANTIS2LIB) in GPS-X. This model is normally recommended following a full influent characterization including manual calculation of the state variables^56^, yet this was not the case in this study due to available influent data taken from publication^51^. However this influent model was found to produce good agreement with the composite variables following an iterative process with only few changes to the default values, including adjusted inputs of 626 mg/L, 44.2 mg/L, 33.7 mg/L and 0.63 mgVSS/mgTSS for total COD, total TKN, ammonia nitrogen (NH_3_) and the VSS/TSS ratio respectively as shown in Table S1 (see Supplementary Material). These changes returned 367.9 mg/L for TSS (4.3% deviation) and 322.9 mg/L (5.4% deviation) for total carbonaceous BOD which remained well within the reported range shown in Table 1.

**Table 1:** Influent, effluent and operational characteristics of HySAF pilot plant in Rishikesh, India used for calibration. Value range provided where present in literature ^51^.

Parameter	Unit	Influent	Effluent
pH	–	7.0 ± 0.6	
Temp	°C	26 ± 4	
COD	mg/L	627 ± 188	62 ± 5
BOD	mg/L	306 ± 84	31
TSS	mg/L	384 ± 80	37
NH_3_	mg/L	33.7 ± 8.2	4.9 ± 1.6
TKN	mg/L	44.2 ± 7.1	6.2
TN	mg/L	46.7 ± 8.5	14.0

These input adjustments held true for the validation influents maintaining a < 5.1% deviation across parameters.

### Aerobic IFAS reactor

The comprehensive model (MANTIS2) was used for this study. This model is a state-of-the-art model that was developed by Hydromantis in order to progress the ASM2d and the anaerobic digester model no. 1 with greater versatility^56^. It was chosen for this study primarily due to its comprehensive approach to total nitrogen (TN) removal which is a key priority in global water management^57^. The hybrid system object was selected which combines the suspended growth model discussed and the GPS-X biofilm model^56^. Kinetic and stoichiometric parameters are not differentiated between colony types (suspended or attached) in this object. User-defined inputs can be found in Table S1 and correspond to the values given by Singh et al.^51^.

### Secondary clarifier

To represent the clarifier a one-dimensional, non-reactive model was used (SIMPLE1D). This model is preferred when biological reactions in the clarifier can be ignored^56^. It divides the sludge blanket into 10 equal layers and assumes the consideration of only vertical flow and that all incoming solids are distributed instantaneously and uniformly across the surface of the feed layer^56^. User defined-inputs can be found in Table S1 and correspond to the values given by Singh et al.^51^.

### Model calibration

Following adjustments of the user-defined values, the steady-state model was run to determine the accuracy of its representation to the published data. The model outputs assessed were BOD, TN and TSS. At this stage the model results, were not in good agreement with the observed results as shown in Fig. S1 (Supplementary Material), so the calibration phase commenced.

### Sensitivity and uncertainty analysis

Sensitivity analyses (SA) are often used by modellers to identify the relative influence that different factors such as parameters and their interactions have on the output of a model^45^. This can aid the modeller in several ways, for instance to simplify models, prioritise parameters for calibration, identify errors in the model and apportion uncertainty between model factors^58^. Parameters that are found to have a small or no influence can be considered negligible and can either be left at default value, given an arbitrary value within the parameter range, or even removed from the model as a form of model reduction, thus simplifying and aiding interpretation without compromising accuracy. The parameters of the greatest influence, or that the model output is most sensitive to, are therefore identified as the parameters that require the most experimental focus during calibration in determining their true values. As the influence of a parameter increases, so does the potential for error which indicates these as the parameters that will require further analysis to quantify the level of uncertainty that they invoke and thus minimize where possible.

A key source of uncertainty in bio-models is derived from the multiple kinetic and stoichiometric parameters that compose the model. These are often difficult, if not impossible to measure due to experimental limitations, and kinetic parameters especially can demonstrate large variability between treatment plants^59^. Despite this, often only a small number of input parameters account for the majority of the uncertainty or variability in model outputs^43^. Once these influential factors have been identified and their share of the uncertainty has been apportioned by the SA, an uncertainty analysis (UA) can be performed to quantify this uncertainty held by each input by observing its propagation through the model. Together SA and UA provide a way to achieve some transparency into the reliability of the model adding (or removing) credibility to conclusions drawn^58^.

For this study, the Python coding platform (Ver 3.7) was used for both the UA and SA, with both SA utilizing the SALib library^60^. The developed code can be found in the supplementary material (Python Scripts S1-S3).

### Method of Morris

The Method of Morris (MOM) is similar to the more commonly used local sensitivity analysis (LSA) in that it only deviates one parameter at a time, however it conforms to GSA definition because the parameter value is started from different points in the possible input space multiple ($$r)$$ times and averaged. While other GSA approaches such as the Sobol method are able to provide a more detailed analysis, these are more computationally-expensive. For example, an analysis with 14 parameters and a sample size of 6,000 would require 6.5 × 10^5^ evaluations^61^. In contrast to the Sobol method, the MOM is able to detect first-order effects at only 100th the computational expense and a 10th of the cost when investigating second order effects^62–65^.

A more efficient approach that is commonly employed is to first use the MOM to screen for non-influential parameters that can be exempt from the Sobol analysis in order to reduce the number of model evaluations needed to obtain adequate decomposition^61,66,67^. Brockmann and Morgenroth ^68^ demonstrated MoM to be just as effective as variance-based methods in distinguishing between influential and non-influential parameters, which was similarly observed by Herman et al.^61^. However in both cases, it was recommended to proceed the more computationally-demanding methods to gain sufficient variance information or better rank the more sensitive parameters. For the purpose of this study the MOM was used as an initial screening of 68 kinetic and stoichiometric parameters that constitute the model to identify the 10 most influential parameters with regards to each model output (BOD, TN, TSS). These parameters were then subjected to further examination under the Sobol analysis. The screened parameters of the investigated model can be found in Table S2.

The MoM uses approximations of the first order partial derivatives of a model called elementary effects (EE) to characterise model sensitivity^69^. In order to determine the EE for each input factor, the differentiation of the model output $y$ is calculated in relation to each input factor ${x}_{i}$ as according to the following equation^69^:

1 $${EE}_{i}= \frac{\delta y}{{\delta x}_{i}}= \frac{y\left({x}_{1}, {x}_{2}, {x}_{i}+ \Delta , \dots {x}_{k}\right)-y\left(x\right)}{\Delta },$$

where ${x}_{i}$ is the $i$th factor of the model, $\Delta$ is the set perturbation factor by which the base value of ${x}_{i}$ is deviated, $y\left(x\right)$ is the model output evaluated at certain nominal values of model factors, $y\left({x}_{1}, {x}_{2}, {x}_{i}+ \Delta , \dots {x}_{k}\right)-y\left(x\right)$ describes the model output corresponding to the predetermined deviation $\Delta$ in ${x}_{i}$.

The finite distribution of the EE, $Fi$, for each factor is obtained by performing $r$ calculations of the EE at independent, randomly sampled points in the input space. This method as described by Morris ^62^ provides $r$ observations of $Fi$ for $k$ factors at a cost of $r$ ($k$ + 1) model evaluations.

In order to determine the mean average of the EE, ${\mu }_{i}$, the following equation is used^69^:

2 $${\mu }_{i}=\frac{1}{r}\sum_{t=1}^{r}{EE}_{i}.$$

While calculation of the standard deviation,${\sigma }_{i}$, is achieved as follows^69^:

3 $${\sigma }_{i}=\sqrt{\frac{1}{r-1}}\sum_{t=1}^{r}{{(EE}_{it}-{\mu }_{i})}^{2}.$$

If the model is non-monotonic, where the variables have a tendency to move in the same direction yet without guarantee of constancy, it is possible for the $Fi$ distribution to return negative values that may cancel positive values and be misrepresented as non-influential. Campolongo et al. ^63^ provided a revision to the method that replaced the mean of the elementary effects, $\mu$, with the absolute mean of the EE, ${\mu }^{*}$. This prevented the effects of opposite signs and provides an index of the magnitude of influence of a parameter by which the overall influence on the output can be ranked accordingly. While it is widely used for screening influential parameters, it is primarily a measure of non-influence^63^. In contrast, the standard deviation of the elementary effects, ${\sigma }_{i}$ considers the variance and detects influence of factor interaction or non-linearity.

In order to calculate the absolute mean of the EE, ${{\mu }_{i}}^{*},$ the following equation was used^69^:

4 $${{\mu }_{i}}^{*}= \frac{1}{r}\sum_{t=1}^{r}|{EE}_{it}|.$$

Parameters are then able to be ranked using the following equation^69^:

5 $$\text{B }= \sqrt{{{\mu }_{i}}^{*2}+ {{\sigma }_{i}}^{2}.}$$

By deriving both the absolute mean and standard deviation of the EE, the effects of investigated parameters can be categorized as follows^69^:

1. Negligible (low average, low standard deviation).
2. Linear and additive (high average, low standard deviation).
3. Non-linear and/or interactions with other parameters (high standard deviation).

In presenting graphical representation of the results from the Morris method, bar charts and Morris plots are both commonly used^66,70^. The Morris plot takes both the ${\mu }^{*}$ and $\sigma$ of the elementary effects (EE) and plots them against each other in a two-dimensional graph. By doing so, parameters with a low mean and low standard deviation will be plotted in the bottom left hand corner and can be considered non-influential^69,70^. Conversely the parameters of influence will be plotted towards the top right hand corner.

Drawbacks of MoM include its tendency to provide only qualitative information by ranking input factors, without quantifying the influence of each factor on the output^66^, as well as its inability to correctly rank the most influential parameters despite being highly effective at separating influential from non-influential parameters^43,61^. Where this method is only to be used as a screening method to reduce computational expense of a further variance-based method such as the Sobol analysis, these limitations become less important.

For this study a sample size (*n*) of 100 was used with the MoM analysis. While previous work has shown a smaller sample size (20 *n*) to be just as effective as higher sample sizes (100 *n*) in differentiating between influential and non-influential parameters even in highly-parameterized models^61^, other work has demonstrated the MoM to neither predict the correct number or designation of influential/non-influential factors in a wastewater biofilm model at lower sample sizes (10–20 *n*) when compared to variance-based methods^71^.

Compared to more comprehensive GSA, Cosenza et al. ^71^ showed this method was unable to converge at lower sample sizes (10–20 *n*) and failed to differentiate between influential and non-influential parameters as effectively. However this is in contrast to work by Herman et al. ^61^ who compared the efficacy of the MoM to differentiate between influential and non-influential parameters across a range of sample sizes. In their study, Herman et al. ^61^ found the MoM to be just as effective in differentiation at 20*n* as 100*n* with little need for higher sample sizes.

### Sobol method

The Sobol method derives the sensitivity indices by attributing variance to single model inputs as first-order Sobol indices (${S}_{i}$), and variance due to interactions between multiple parameters as higher-order Sobol indices^45^. A second-order effect (${S}_{ij}$) is characterised by an interaction between two parameters, while a third-order effect relates to an interaction between three parameters and so on. When ${\sum }_{i}{S}_{i}\ne 1,$ the presence of interactions are indicated as well as their influence relative to ${S}_{i}$^45^. Total-order Sobol indices (${S}_{Ti}$) are determined as the sum of apportioned variance for any parameter and its interactions. In linear models the sum of ${S}_{Ti}$ should equal 1 while in non-linear models the sum should exceed 1^45^. When ${S}_{Ti}$ is observed to be substantially higher than ${S}_{i}$ for any given factor, it is indicative of a higher order interaction occurring with other parameters. Over-parameterised models can therefore be reduced by discounting parameters that demonstrate a low ${S}_{Ti}$ as these can be assumed to hold negligible influence due to the inclusiveness of this index^66^.

In this study the Sobol indices (SI) were calculated through random sampling (*n* = 10,000) by way of Monte Carlo simulation (MCS). Four steps were taken to apply the Sobol procedure as described by Ref.^72^: 1—The uncertainty ranges for each input parameter were defined as ± 50% of the default value in GPS-X, 2—Sobol sampling was used to sample the range of each parameter, 3—uncertainty was propagated through the model by repeated simulations for each combination of the input parameters within their ranges, 4—acquired data was post-processed to calculate first-order SI, second-order SI, and total-order SI as follows^73^:

6 $${\text{First{-}order SI}, S}_{i}=\frac{{V}_{i}}{V},$$

7 $${\text{Second{-}order SI}, S}_{ij}=\frac{{V}_{ij}}{V},$$

8 $$\text{Total{-}order SI}, {S}_{Ti}= {S}_{i}+ {\sum }_{j\ne i}{S}_{ij},$$

where the partial variance, ${V}_{i}$ = $V[E\left(Y|{X}_{i}\right)]$ which is the variance of the conditional expectation, held over the unconditional variance $V$ with ${X}_{i}$ representing the input parameters and $Y$ the model output or objective function^73^. The total contribution of variance of each input parameter ($p$) and interactions can thus be determined by the following decomposition:

9 $$V\left(Y\right)= {\sum }_{i=1}^{P}{V}_{i}+ {\sum }_{i=1}^{p-1}{\sum }_{j=i+1}^{p}{V}_{ij}+\dots + {V}_{1,\dots ,p}.$$

The role of the Sobol method in automatic calibration is to test for possible collinearity and reduce the calibration effort of a potentially over-parameterized model by differentiating between parameters of influence and non-influence. While this may be apparent, it is appropriate to define the level of influence required to warrant calibration and signify collinearity. In this study a threshold of 0.05 was defined as used in previous work^42^.

### Distinguishing between influential/non-influential parameters

Following each of the GSAs, it was necessary to distinguish between influential and non-influential in order to reduce the model for a more focused calibration. Following the MoM analysis, the output values for each parameter were first normalised within the 0–1 range. By normalizing the data, the capacity of the MoM to identify influential parameters could then be compared against that of the more-detailed Sobol analysis once thresholds of influence have been defined ^74,75^. With regards to the MoM, both Ramin et al. ^74^ and Valverde-Pérez et al. ^76^ applied a threshold of 0.1 ${\mu }^{*}$, while Hsieh et al. ^75^ used thresholds of 0.1 ${\mu }^{*}$ for the MoM and 0.05 for the Sobol analysis to good effect. Previous work by Zhang et al. ^42^ also proposed an influence threshold of 0.05 for the Sobol analysis, with this value requiring an influential parameter to account for at least 5% of the variance. In line with these past definitions, a threshold of 0.1 ${\mu }^{*}$ was used for the MoM and 0.05 for the Sobol analysis.

Data was normalised using the following equation:

10 $${z}_{i}= \frac{({x}_{i}-{x}_{min})}{({x}_{max}-{x}_{min})},$$

where ${z}_{i}$ is the ${i}_{th}$ normalized data set and ${x}_{i}=({x}_{1}, .. , {x}_{n})$.

### Parameter estimation for calibration

A computational optimization exercise was performed for the purpose of estimating the kinetic and stoichiometric parameters of greatest influence by means of the maximum likelihood function. Further details of the maximum likelihood function utilized can be found in Hydromantis^56^.

With process models typically being of a non-linear nature, analytical determination of the their optimized values are often not possible. To combat this, a derivative-free optimization method, namely the Nelder and Mead (NM) simplex method ^77^ was employed. This method was first proposed by Spendley et al. ^78^ before being better refined by Nelder and Mead ^36^ to the core form that has now been used for over half a century due to its many advantages. It can be considered a direct line-search method of steepest descent exploration, due to its system of searching the factor space. The method makes use of a polyhedron of *N* + 1 sides (*N* = number of input variables) that systematically reflects, expands, contracts and shrinks to explore the factor space for a minimum^36,79^. On every iteration the function values are taken from the vertices and the highest value will be discarded and a new point would be sought in the general direction of a negative gradient.

The NM method offers multiple advantages over derivative-based alternatives due to its robust approach to optimization. Its simplicity to implement and understand without the need for any derivative information make it an attractive option for engineers. While derivative-based methods tend to be faster in returning a result^79^, they can also be vulnerable to noise in the function values while the NM method here demonstrates a high tolerance. Additionally the NM method thrives in more complex terrain of the objective function due to its capacity to rapidly adjust its shape, size and orientation based on local contours^79^. While it has faced some criticism from mathematicians due to its inability for convergence to be proven^80^, it has stood the test of time with engineers for its many examples of successful optimization, particularly in parameter estimation^81,82^.

In estimating the values of the kinetic and stoichiometric parameters as part of the calibration, the current task was defined as a multi-objective, constrained optimization problem with the purpose of minimizing the (negative) error between the target values of objectives (effluents: BOD, TN and TSS) and the model output for these parameters. In this way the maximum likelihood function can be maximized as required to determine the optimal parameter estimates by using a minimizer^56^. The error distribution was considered normal and termination criteria was set as no further significant change in parameters.

### Model validation

Model validation is a necessary part of model development. As defined by Schlesinger et al. ^83^, “model validation is the substantiation that a computerized model within its domain of applicability possesses a satisfactory range of accuracy consistent with the intended application of the model”. Put more simply, it seeks to assess whether a model is of an acceptable accuracy for its intended purpose^84^.

In the present work, data derived from alternative DO regimes were used (0.5 mg/L, 4.5 mg/L) from a previous experiment as shown in Table 2^51^. This was considered sufficient in terms of technical rigour because separate influent and effluent data was provided conforming to the need for independent data to be used in validation^85^. A similar approach to calibration and validation has been used in previous work^86^. This also provided a means for validity to be assessed at two separate points as opposed to the single-point validation that is more traditional^85^.

**Table 2:** Influent, effluent and operational characteristics of HySAF pilot plant in Rishikesh, India used for validation. Value range provided where present in literature^51^.

Parameter	Unit	0.5 mg/L DO regime		4.5 mg/L DO regime	
		Influent	Effluent	Influent	Effluent
pH	–	7.2 ± 0.2		7.2 ± 0.2	
Temperature	°C	26.5 ± 1.5		23 ± 2.0	
Chemical oxygen demand (COD)	mg/L	455.6 ± 37.1	85.0	440.4 ± 25.7	25.5
Biochemical oxygen demand (BOD)	mg/L	232.8 ± 29.8	46.0	221.6 ± 18.4	9.0
Total suspended solids (TSS)	mg/L	316.6 ± 34.7	58.0	262.9 ± 27.6	15.0
Ammonia (NH_3_)	mg/L	40.8 ± 7.9	32.8 ± 14.3	34.5 ± 9.6	0.2 ± 0.2
Total Kjeldahl nitrogen (TKN)	mg/L	48.6 ± 7.6	35.6	42.5 ± 9.6	1.3
Total nitrogen (TN)	mg/L	50.7 ± 8.6	43.6	45.9 ± 11.6	14.2
Hydraulic retention time (HRT)	h	11.1		11.1	
Solids retention time (SRT)	days	8		22	
Bulk dissolved oxygen (DO)	mg/L	~ 0.5		~ 4.5	
Mixed liquor suspended solids (MLSS)	mg/L	2000 ± 200		2000 ± 200	
(RAS)	x Q	2.5		3.0	

### Uncertainty analysis

Following model calibration, an uncertainty analysis was carried out to quantify the uncertainty surrounding the determined values of parameters deemed to be influential^48^. For this a Monte Carlo simulation (MCS) was used. MCS is commonly used for this purpose due to its robustness as it accounts for non-linearity in the model and correlation where specified while being more accessible in terms of mathematical intensity compared to alternative techniques^87^. Furthermore, it naturally provides a graphical representation of the output distribution which can aid identification of skewed or non-normal distribution in the measured^87^. Farrance and Frenkel ^87^ provide a detailed comparison between this approach and older standards such the guide to the expression of uncertainty in measurement (GUM) method.

In applying the MCS, a probability distribution is first prescribed for the input parameters being investigated. A key source of uncertainty in the model output can come from a lack of information regarding the precise value that the input should hold. Where there is variability in this value, for example between WWTP, the value can be represented by a probability distribution. When this distribution is known, the uncertainty can be reduced as a more accurate estimate of the true value can be assigned in lieu of precise knowledge while the uncertainty held in this estimation can be better evaluated.

Unfortunately it is often the case that the probability distribution is unknown and in place of such information expert judgement needs to be assigned^88^. For example, where estimates can be made regarding the likely range of the parameter, but no information regarding the distribution, a uniform distribution can be assigned. When information is available regarding occurrence of values around the mean and experimental bias is known to not be influencing results, a normal distribution can be used^88^. The use of a uniform PDF reflects the probability of obtaining a value anywhere between the defined upper and lower limits. This PDF is usually chosen when knowledge on the distribution is little and only the limits are known^87^. This is the most conservative estimate of uncertainty since it leads to the largest value.

Once the distribution has been assigned to each of the investigated model inputs based on limits and shape with the capture of the real value assured, pseudo-random samples are taken from within each of these distributions and the model is evaluated based on these de facto input parameters. The corresponding functional output is then reported and can be graphically represented to present the probability distribution function (PDF) of the investigated outputs. The larger the number of samples taken, the more accurately the standard uncertainty in the inputs can be represented and propagated through the system. However as shown by Farrance and Frenkel^87^, the difference in variability between sample sizes will reduce considerably at higher orders of magnitude. While a sample size of 1 × 10^3^ was observed to return a similar estimation of the standard uncertainty in the parameter, a sample size of 1 × 10^5^ offered improved numerical precision and consistency.

Following determination of the PDF the required information can be calculated by way of simple mathematical procedures from the output data. The mean average of the returned values, $\overline{x }$, gives the calculated estimate of the parameter’s true output value, while the standard deviation, $S$, indicates its standard uncertainty^87^. Accurate calculation of $S$ will depend on the assigned shape of the parameter’s PDF with each shape having its own equation:

11 $$\text{Standard deviation for a uniform distribution}, {S}_{uni}= \frac{a}{\sqrt{3}},$$

12 $$\text{Standard deviation for a normal distribution}, {S}_{norm}= \sqrt{\frac{\sum ({{x}_{1}-\overline{x })}^{2}}{n-1},}$$

13 $${\text{Standard deviation for a triangular distribution}, S}_{tri}= \frac{a}{\sqrt{6}},$$

where $a$ is the ± limit from $\overline{x }$ or the half-width of the distribution. The appropriate PDF assignment for each investigated parameter will depend on the nature of the data in terms of its limits and where the values are likely to fall.

A coverage value, $k,$ of 1.96 is used in order to derive the expanded uncertainty, ${U}_{e}$, of within a 95% coverage interval as follows^87^:

14 $${U}_{e}=1.96\left({U}_{c}\right)\text{ for a }95\text{\% coverage interval}.$$

## Results

### Model calibration and validation

#### Sensitivity screening phase

**Figure 2:**
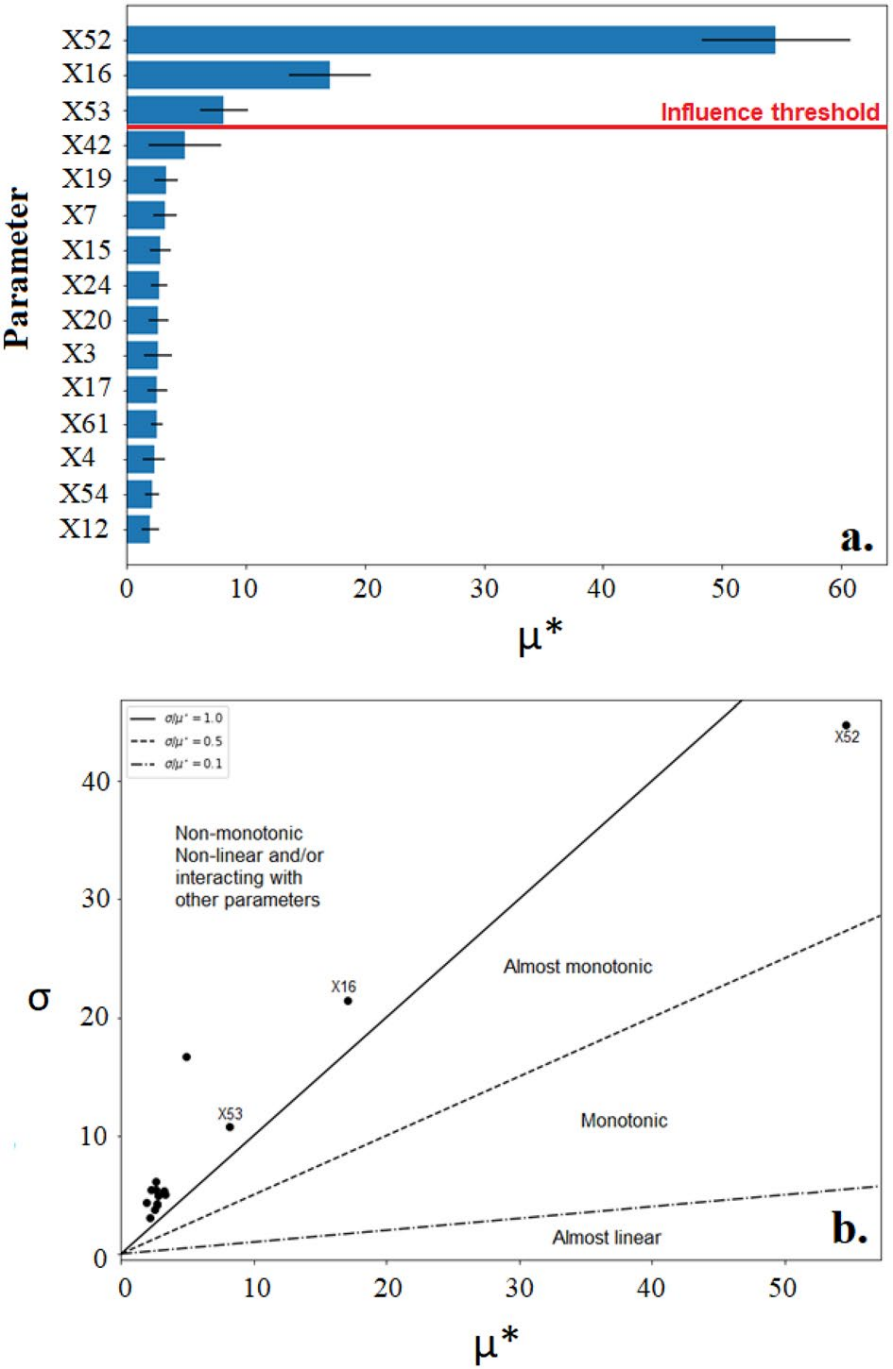
The 15 parameters of greatest influence on the BOD output as demonstrated by the MoM displayed by (**a**) A horizontal bar chart with influence threshold 0.1 and (**b**) a Morris plot (σ/μ*).

Results from the screening phase of the calibration process attempted to distinguish between influential and non-influential parameters. As shown in Fig. 2a, three parameters are suggested as influential on the BOD model including X52 (aerobic heterotrophic yield on soluble substrate), X16 (aerobic heterotrophic decay rate), and X53 (anoxic heterotrophic yield on soluble substrate) being ranked by μ* scores in this order. Figure 2b displayed the presence of a monotonic relationship between X52 and the model output, implying a strong correlation and a focal parameter for calibration. The dominant influence of this parameter is unsurprising as it reflects the critical role that ordinary heterotrophic organisms (OHOs) take in the degradation of waste organic matter by way of receiving the organic compound’s electrons and utilizing the carbon for cell synthesis^89^. The importance of accurately portraying the actual proliferation of OHOs in both aerobic and anoxic environments of any given wastewater system has long been recognised^90^.

**Figure 3:**
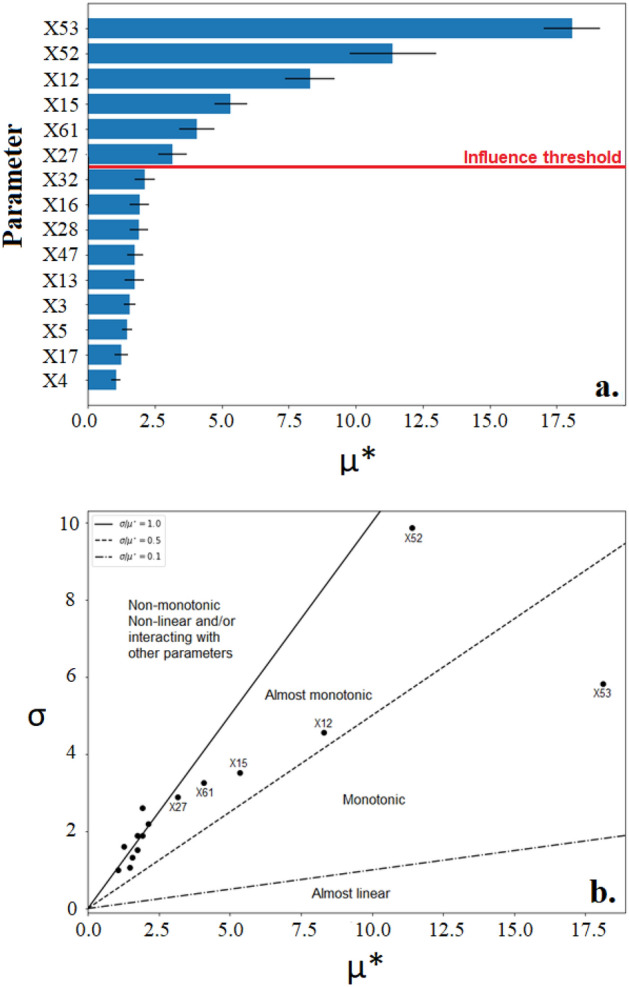
(**a**) The 15 parameters of greatest influence on the TN output as demonstrated by the MoM displayed by (**a**) A horizontal bar chart with influence threshold 0.1 and (**b**) a Morris plot (σ/μ*).

The TN removal model was found to be sensitive to a greater number of parameters than the BOD removal with 6 parameters breaching the threshold influence (0.1) following normalization as shown in Fig. 3a. Amongst the influential parameters, X53 and X52 incurred the greatest sensitivity respectively, with the former demonstrating the greatest linearity as shown in 3b. These two parameters have previously been identified as strongly influential on AS models ^90^ but their value as key parameters in IFAS models also are here supported. TN removal models of other bioreactors such as the membrane bioreactor (MBR) have also been found as most sensitive to the anoxic heterotrophic yield parameter which further emphasizes its critical role in effective simulation^91^. Other parameters suggested to be influential on TN removal included X12 (reduction factor for denitrification on nitrite-N), X15 (oxygen inhibition coefficient for denitrification), X61 (unbiodegradable fraction from cell decay) and X27 (maximum growth rate for nitrite oxidizer) in this order.

**Figure 4:**
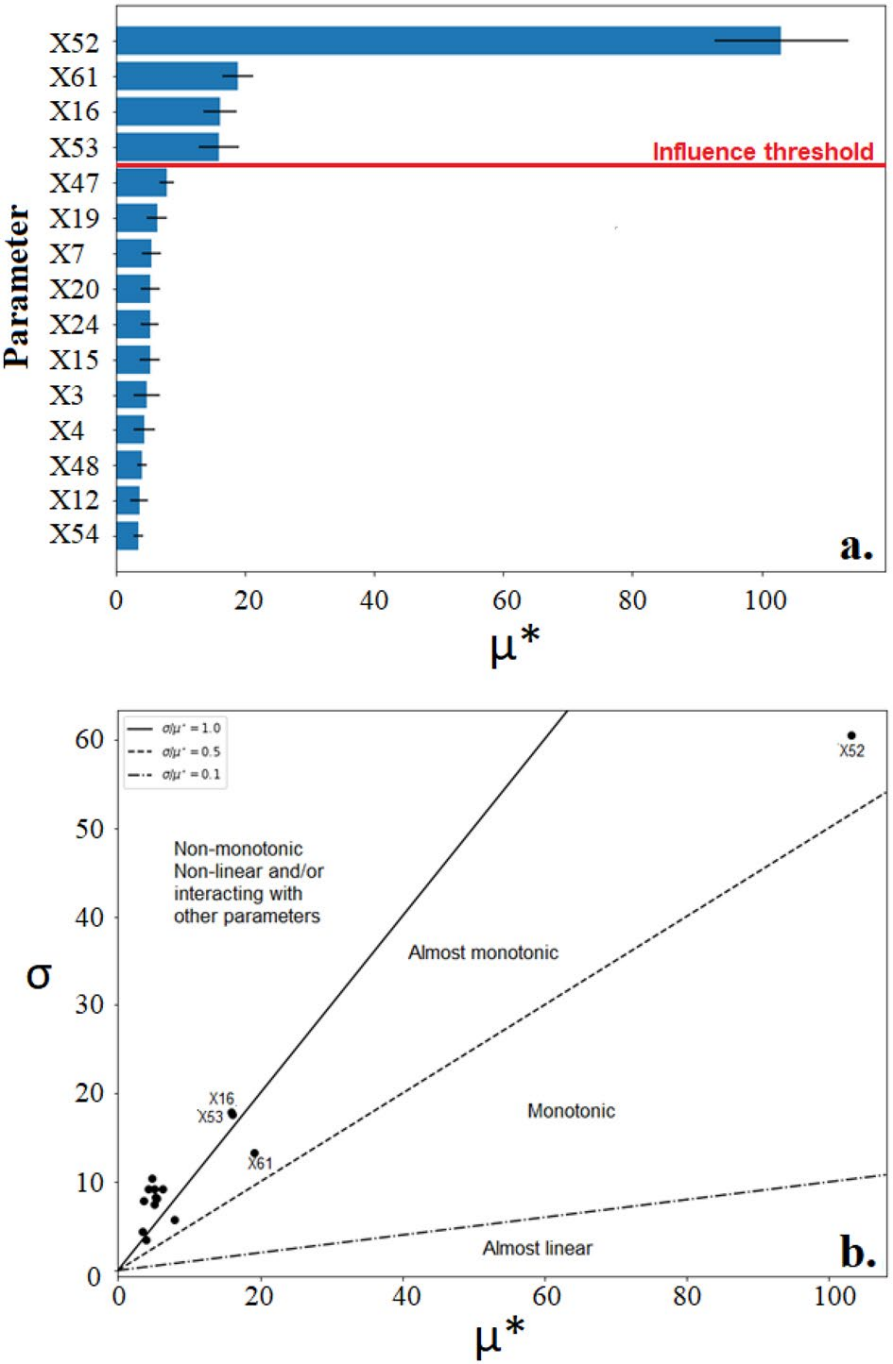
(**a**) The 15 parameters of greatest influence on the TSS output as demonstrated by the MoM displayed by (**a**). A horizontal bar chart with influence threshold 0.1 and (**b**) a Morris plot (σ/μ*).

In terms of TSS removal, Fig. 4a shows four parameters were suggested as influential including X52, X61, X16 and X53 in this order. Figure 4b displays a strong asymmetry between the parameters identified as influential with X52 as a clear outlier. This is not surprising as heterotrophic bacteria are the dominant type in AS systems ^92^ and have been shown to positively influence settling velocities of AS flocs more than nitrifiers^93^.

The MoM identified multiple parameters to be influential across more than one model which warranted greater insight into their relative effect on each model. Table 3 showed that X52 and X53 were influential on each of the three models, while X61 and X16 were influential to two. Besides identifying the parameters with a broader influence, Table 3 also identified parameters with conflicting polarity. For example, this was apparent for X52. In this instance, any adjustment of this parameter to reduce error between actual and simulated output during calibration of the BOD and TSS models would increase error for the TN model and vice versa. As such X52 was shown to have an inverse monotonic relationship with the TN model output in contrast to the direct monotonic relationship of the other models. The polarity of other influential parameters was not seen to differ across models.

**Table 3:** Models sensitive to parameters identified as influential by MoM with polarity.

Code	Parameters	Sensitive models		
		BOD	TN	TSS
X52	Aerobic heterotrophic yield on soluble substrate	+	−	+
X53	Anoxic heterotrophic yield on soluble substrate	+	+	+
X61	Unbiodegradable fraction from cell decay		+	+
X16	Aerobic heterotrophic decay rate	−		−
X12	Reduction factor for denitrification on nitrite-N		−	
X15	Oxygen inhibition coefficient for denitrification		−	
X27	Maximum growth rate for nitrite oxidizer		+	

## Variance-based sensitivity analysis

**Figure 5:**
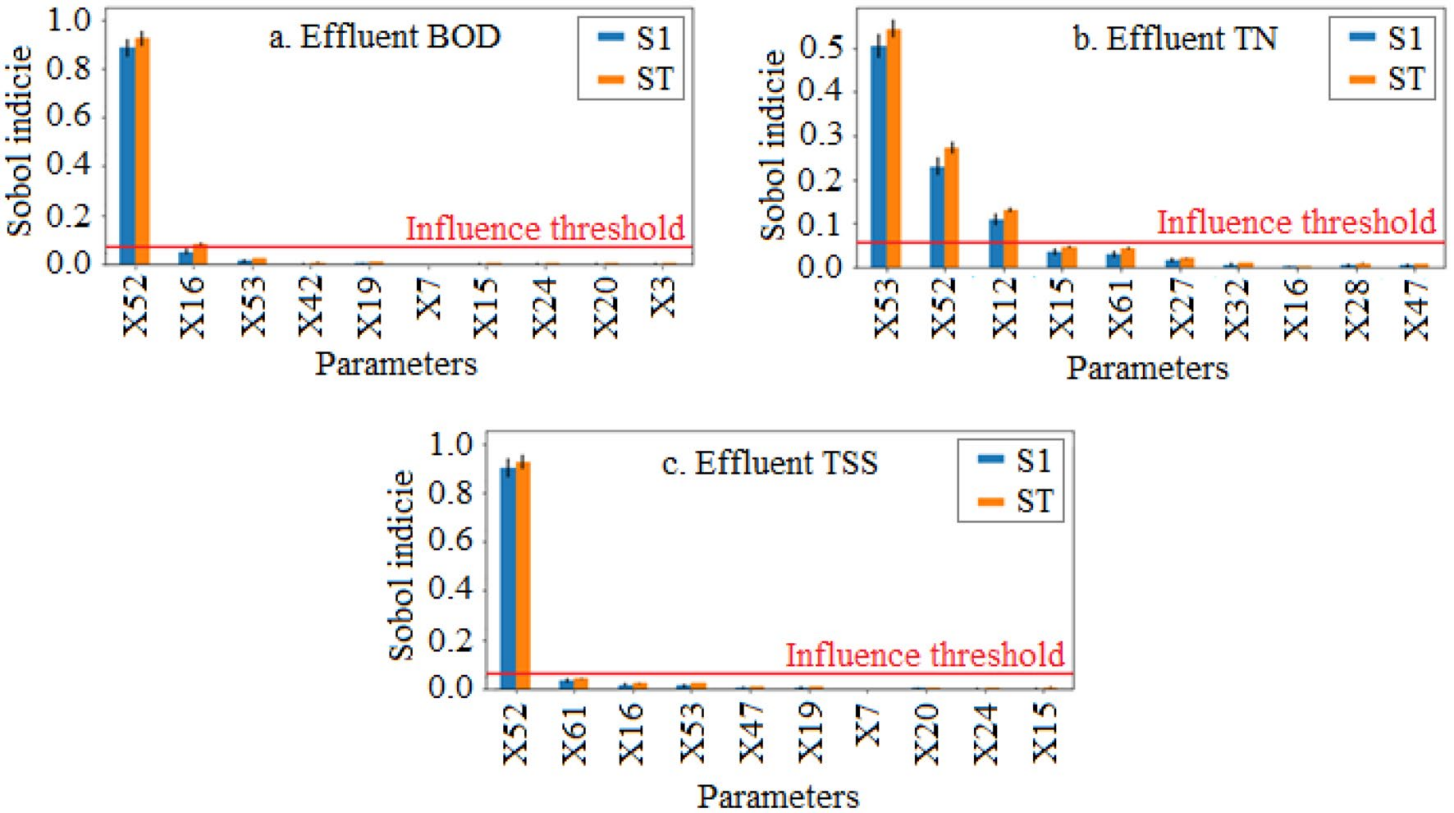
Top 10 parameters ranked according to influence for (**a**) BOD model, (**b**) TN model, (**c**) TSS model with influence threshold displayed (0.05).

In comparison to the MoM, the Sobol analysis was seen to be more selective of influential parameters at the given threshold (SI < 0.05). Table 4 identifies only four parameters to meet the influence criteria designated by Zhang et al.^42^. Of these parameters only X52 was observed to significantly influence all three models, while the remaining parameters only influenced one model each. In terms of variance, X52 accounted for 92.4% of the variance in the BOD model, 27.3% in the TN model and 92.8% in the TSS model. In each case this was mainly attributed to the first order effects as shown in Fig. 5. X53 and X12 were only influential to the TN model and accounted for 54.3% and 13.1% of the variance respectively, again due mainly to the first order effects. Finally, X16 only had significant influence on the BOD model and accounted for 8.0% of the total variance, while the first order effects only accounted for 5.0%.

**Table 4:** Parameters identified as influential by the Sobol analysis (> 0.05) with observed Sobol indices for first and total order effects.

Code	Parameters	BOD model		TN model		TSS model	
		S1	ST	S1	ST	S1	ST
X52	Aerobic heterotrophic yield on soluble substrate	0.885	0.924	0.230	0.273	0.904	0.928
X53	Anoxic heterotrophic yield on soluble substrate	–	–	0.505	0.543	–	–
X16	Aerobic heterotrophic decay rate	0.050	0.080	–	–	–	–
X12	Reduction factor for denitrification on nitrite-N	–	–	0.109	0.131	–	–

All results displayed demonstrate a 95% confidence level.

**Figure 6:**
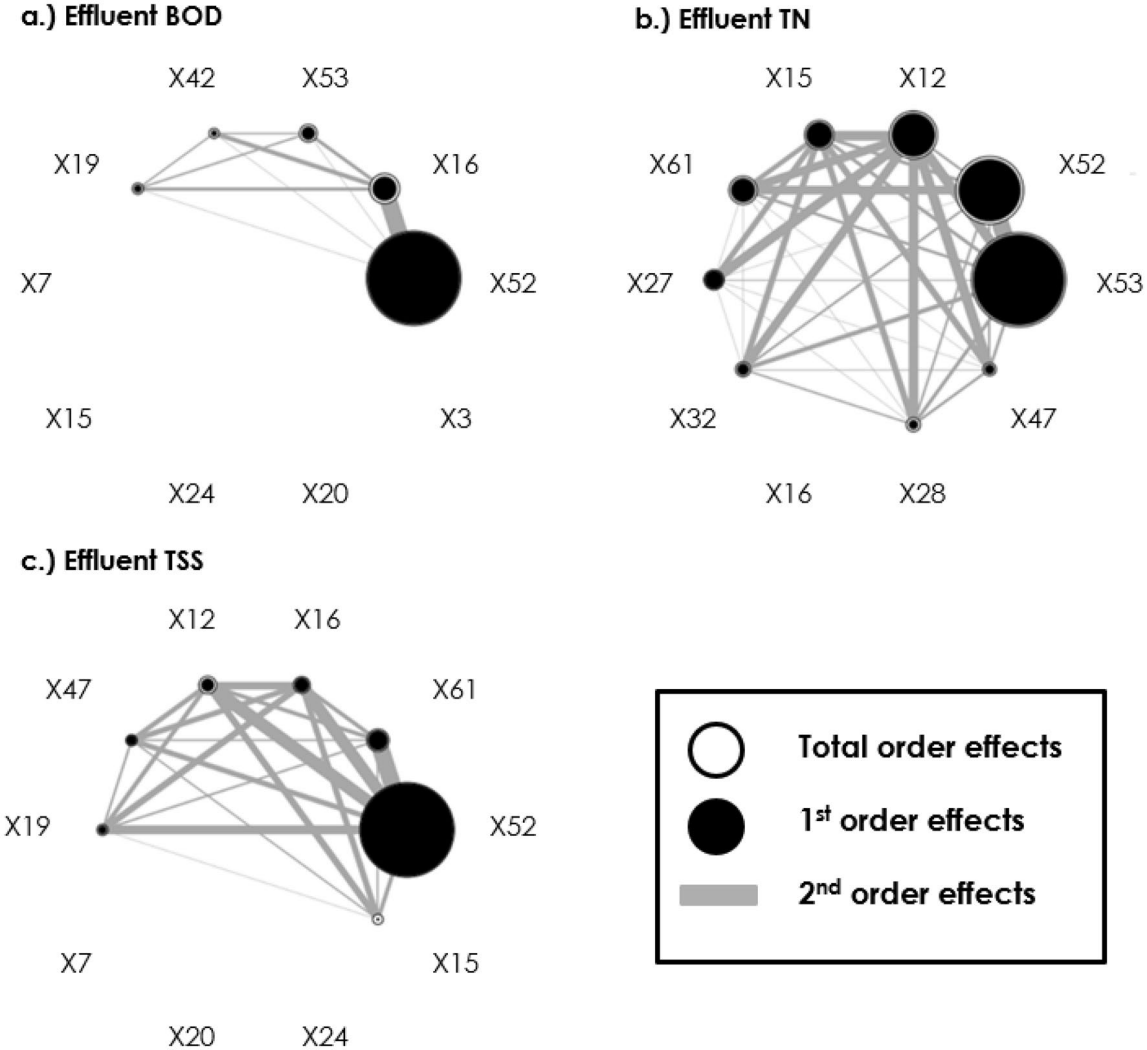
Second-order interactions and relative influence of the first and total order effects of investigated parameters on (**a**) BOD, (**b**) TN, and (**c**) TSS models.

The Sobol analysis also identified minimal presence of higher order effects for each of the parameters. Despite the presence of multiple interactions between parameters as shown by their communication in Fig. 6, these remained below the given threshold (SI < 0.05). This was supported by the absence of substantial white rings around each parameter that would indicate a greater influence of higher-order effects relative to the first order. This suggest that non-linearity is not a significant factor in the present model. This may be attributed to its steady-state nature, as well as lacking wide factor variability ranges and additional recycle streams that have otherwise been proposed as causal reasons for increased detectability of parameter interactions^94^.

What is also apparent from the Sobol analysis is the greater interactivity of factors in the TN model compared to the BOD model as shown in Fig. 6. While no interactions were found to be significant in terms of the threshold, relatively speaking X12 was observed to have a greater symmetry of influence across interacting parameters than alternatives including the more influential parameters. This may be due to its role in governing the prominent pathway for TN removal that would be expected to offer broad influence^95^. In contrast, the BOD model demonstrates only one interaction of relative importance, relating the yield and decay of aerobic heterotrophs that are central to BOD removal^96^.

### Parameter estimation for calibration

The Nelder–Mead algorithm was used to estimate appropriate values for identified parameters of influence. Estimated values are reported in Table 5. The stoichiometric parameters, X52 and X53, required only minor adjustments from default within ± 0.07 mgCOD/mgCOD for each. This was to be expected given the greater observed sensitivity of the model to these parameters, while still conforming to previously advised values determined experimentally^97^. While the kinetic parameters, X16 and X12, observed greater deviation from default with ± 0.11 1/day and ± 0.09 (–) respectively, the disparity remained marginal. Previous work by Li et al. ^98^ found the physical properties of the biofilm to be of greater influence than kinetic parameters when modelling IFAS systems, which may explain the need for a minimal adjustment.

**Table 5:** Default and estimated values for identified influential parameters.

Code	Parameter	Unit	Default value	Estimated value
X52	Aerobic heterotrophic yield on soluble substrate	mgCOD/mgCOD	0.666	0.65
X53	Anoxic heterotrophic yield on soluble substrate	mgCOD/mgCOD	0.533	0.52
X16	Aerobic heterotrophic decay rate	1/day	0.62	0.51
X12	Reduction factor for denitrification on nitrite-N	–	0.48	0.39

In contrast, Shaw et al. ^99^ were required to adjust two non-physical parameters beyond reasonable limits in their study (± 3.2 units) to sufficiently simulate simultaneous nitrification denitrification (SND) behaviour in an IFAS system. In their study, the anoxic oxygen half-saturation coefficient for heterotrophs and the maximum specific hydrolysis rate parameters required considerable deviation in order to capture NO_3_ dynamics which was still unable to be fully achieved. This highlights the difficulty of simulating nitrification dynamics effectively. Previous work has highlighted the importance of correctly characterising the nitrifiers when modelling IFAS systems^98^.

Other work has suggested that only minor adjustments to default parameters are necessary^6,40,100^. Hulsbeek et al. ^6^ suggested that any substantial deviation from default parameter values tends to be indicative of a misrepresentation of the actual operational parameters that deserve particular attention. Work by Meijer et al. ^40^demonstrated the greater importance of operational parameters compared to kinetic parameters in terms of influencing ASM outputs, while Schraa et al. ^100^ found successful calibration of an IFAS model in GPS-X to only require minor adjustments.

**Figure 7:**
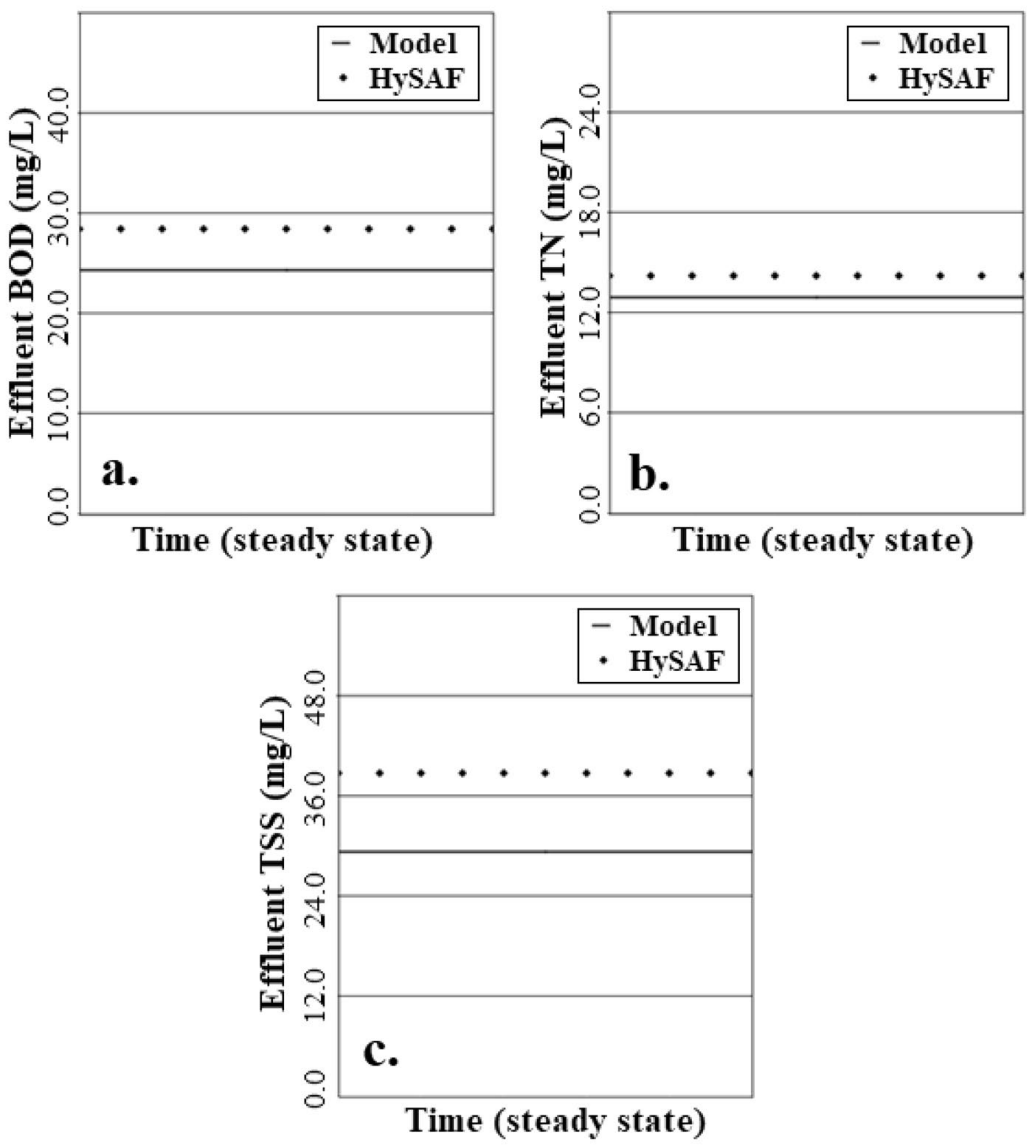
Achieved accuracy of the parameter estimation phase for (**a**) BOD model, (**b**) TN model, and (**c**) TSS model.

The use of the estimated values derived by the algorithm brought model outputs into good agreement with the pilot plant as shown in Fig. 7. The model yielded effluent concentrations of 24.8 mg/L, 12.9 mg/L and 29.5 mg/L for BOD, TN and TSS respectively. This was in contrast to the observed average effluent concentrations of the HySAF which were reported as 28.4 mg/L, 14.2 mg/L and 38.7 mg/L for BOD, TN and TSS respectively^51^. While the model had a tendency to underpredict the HySAF system, this was considered to be within an acceptable limit of deviation with ± 3.6 mg/L, ± 1.3 mg/L, and ± 9.5 mg/L for BOD, TN and TSS respectively.

### Model validation

**Figure 8:**
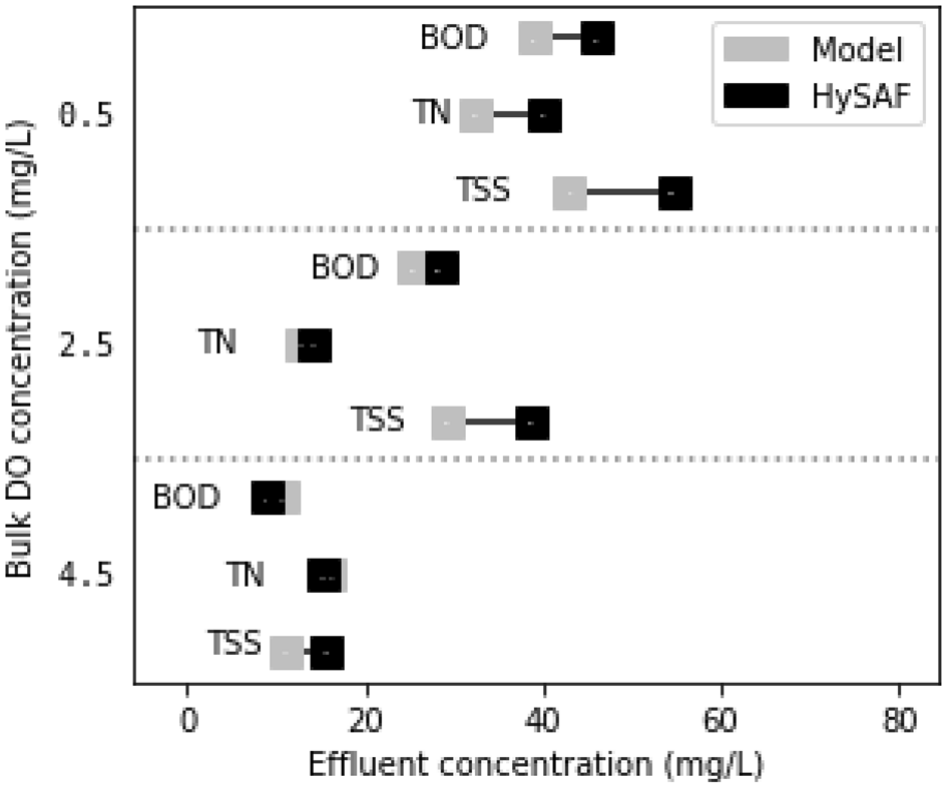
Accuracy of model calibration and validation with regards to effluent BOD, TN and TSS.

The accuracy of the calibration was validated at alternative DO concentrations. As shown in Fig. 8, the validation showed the calibrated model to be in good agreement with reported results. In terms of TN removal, the model was found to offer adequate representation of pilot results at both the calibration and higher DO level. Singh et al. ^51^ had found both these DO concentrations to maintain a similar removal efficiency, ensuring effluent TN concentrations within 10–20 mg/L. However, at the 0.5 mg/L DO concentration the model overpredicted the performance of the pilot plant, achieving an effluent concentration of 32.4 mg/L compared to the 40.4 mg/L. One reason for this may be the wider range of influent TN reported for the lower regime in the pilot study fluctuating between 32 and 62 mg/L compared to 33–55 mg/L at the calibration level^51^.

With regards to the model’s capacity to predict BOD removal of the pilot system, a similar trend was observed whereby the model was found to be in good agreement at the calibration and higher DO regime within 3.6 mg/L difference. However, at the lower DO regime the model was again seen to overpredict the systems performance by 6.8 mg/L. This was also observed to be true for TSS, with a more accurate representation of plant performance at the higher DO setting. Across DO regimes, TSS saw the greatest relative disparity when compared to the other two effluent parameters.

The overprediction of the model at the lowest settings, may reflect the difficulty of understanding IFAS behaviour at such low DO concentrations. Low DO availability coupled with high carbon loading is known to be a favourable environment for filamentous bacteria proliferation that will be detrimental to sludge settleability^101,102^. Organics removal would also be affected adversely under such conditions, with this process being primarily achieved through sludge wastage as well as through metabolism by aerobic heterotrophs^103^. In terms of TN removal, this low of an oxygen level is substantially below concentrations required to achieve nitrification in IFAS^98,104^. However, recent work has suggested that ammonia may still be reduced through unconventional pathways when under such DO-limited conditions^105,106^. While this may be true, the rate of ammonia reduction in the present system remains overpredicted at this DO setting.

Overall, this validation has demonstrated that the model provides a capable simulation at DO levels suitable for IFAS system operation, but may not be appropriate for predicting behaviour under extreme DO stress.

### Uncertainty analysis

Parameters of interest were the four parameters whose total order effect reached the influence threshold of 0.05 during the Sobol analyses^42^. These parameters were the two stoichiometric parameters, aerobic and anoxic heterotrophic yield on soluble substrate, as well as the two kinetic parameters, heterotrophic decay rate and reduction factor for denitrification on NO_2_.

In order to determine the standard uncertainty of each of the influential parameters, including the appropriate PDF that will form the basis of the uncertainty analysis the literature was consulted according to Table 6. Based on these literature values, the ranges of uncertainty for each parameter were taken as 0.63–0.69 mgCOD/mgCOD for X52, 0.52–0.57 mgCOD/mgCOD for X53, 0.23–0.7 d^−1^ for X16 and 0.375–0.48 for X12 assuming equal reduction for NO_2_ and NO_3_.

**Table 6:** Literature values for kinetic and stoichiometric parameters deemed influential (> 0.05).

Symbol	Characterization	Unit	Literature value	References
X52	Aerobic heterotrophic yield on soluble substrate	mgCOD/mgCOD	0.67	^1,3,90,97,107–109^
			0.68–0.69	^110,111^
			0.63	^112,113^
X53	Anoxic heterotrophic yield on soluble substrate	mgCOD/mgCOD	0.52–0.57	^90,97,107–109,114^
			0.53	^115^
X16	Aerobic heterotrophic decay rate	d^-1^	0.28–0.76	^116^
			0.38	^96^
			0.35	^117^
			0.23	^111^
			0.2	^109,118–120^
			0.24	^112,121^
			0.21	^122^
			0.4	^123^
X12	Reduction factor for denitrification on NO_2_	–	0.48	GPS-X default value
			0.8*	GPS-X default value
			0.8*	^112^
			0.75*	^124^

*Total reduction factor for denitrification (NO_2_ and NO_3_)*.*

**Figure 9:**
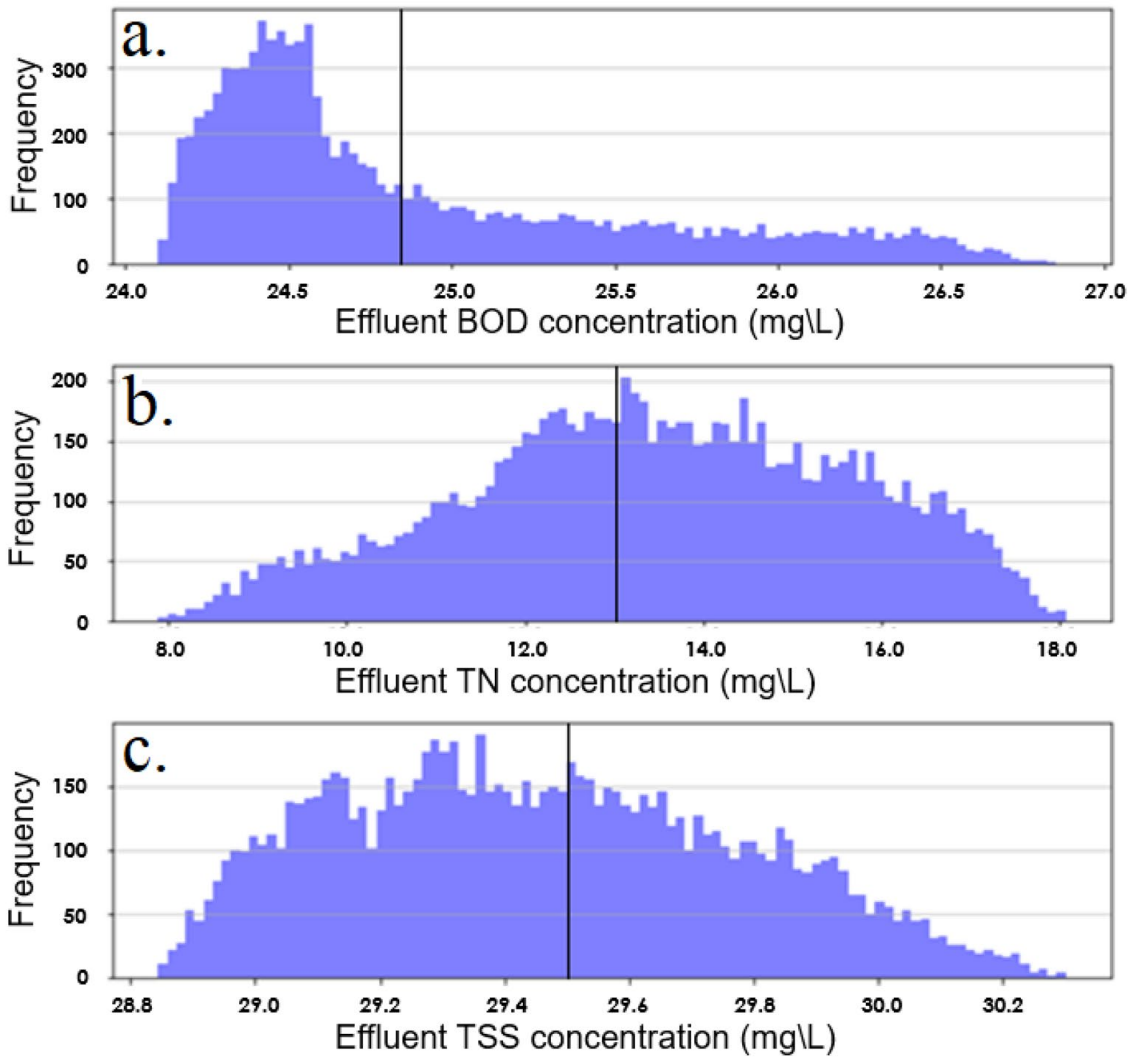
PDFs of combined standard uncertainty analysis and central value (solid line) with regards to (**a**) BOD model, (**b**) TN model and (**c**) TSS model.

Based on these derived ranges, the combined standard uncertainty could be calculated for each of the three models as shown in Fig. 9. A uniform distribution was assumed for each parameter as there was no reason to believe a greater probability of the values localizing within the defined limits^87^.

Following this, the model delivered an effluent BOD concentration of 24.8 ± 1.31 mg/L (95% coverage interval) based on a combined standard uncertainty of 0.67 mg/L. The effluent TN concentration was reported as 12.9 ± 4.23 mg/L (95% coverage interval) based on a combined standard uncertainty of 2.16 mg/L. Finally, the effluent TSS concentration could be reported as 29.5 ± 0.64 mg/L (95% coverage interval).

The TN model was shown to carry the greatest uncertainty (± 4.23 mg/L) representing the sensitivity of the model to a broader range of both influential and non-influential parameters as shown in Fig. 5b that will each propagate a degree of uncertainty through the model. This uncertainty may be reduced further through deeper investigation of the relative contribution of biochemical processes from the attached and suspended colonies that has been suggested as critical in IFAS models^125^. This will vary largely from system to system with the relative contribution of each likely to be governed by many factors including biofilm thickness, aerobic mixing, sheer rate, MLSS concentration, temperature to name a few^126–128^.

## Conclusion

This work has demonstrated the plausibility of combining GSAs with the NM simplex algorithm as a means of calibrating a steady-state biological wastewater treatment model. Simulation of the investigated system while operated at different DO set points provided suitable validation, while also identifying limitations of the model to predict system behaviour under conditions of extreme DO stress.

Of the investigated parameters, only four were found to significantly influence the model including aerobic heterotrophic yield on soluble substrate, anoxic heterotrophic yield on soluble substrate, aerobic heterotrophic decay rate and reduction factor for denitrification on NO_2_. Of these, the stoichiometric parameters were shown to be most influential but in all cases influence was attributed mainly to the first order effects with no considerable collinearity detected.

Parameter estimation by the NM algorithm identified only minor adjustments were required to influential parameters for the model to predict actual system outputs with sufficient accuracy.

Finally, parameter uncertainty was observed to be minimal for the BOD and TSS models, however the TN model demonstrated greater uncertainty that may warrant further work to support the drawn conclusions.

It is hoped that the results of this calibration will inform the future development of steady-state IFAS models. The efficacy of this approach in calibrating dynamic WWT models should also be investigated.

## Supplementary Information

Supplementary Information.

## Data Availability

The datasets used and/or analysed during the current study are available from the corresponding author on reasonable request.

## References

[1] Henze M, Grady CL, Gujer W, Marais GVR, Matsuo T (1987). A general model for single-sludge wastewater treatment systems. Water Res..

[2] Gernaey KV, van Loosdrecht MC, Henze M, Lind M, Jørgensen SB (2004). Activated sludge wastewater treatment plant modelling and simulation: state of the art. Environ. Model. Softw..

[3] Sin G (2005). A new approach for modelling simultaneous storage and growth processes for activated sludge systems under aerobic conditions. Biotechnol. Bioeng..

[4] Hauduc H (2009). Activated sludge modelling in practice: An international survey. Water Sci. Technol..

[5] Hauduc H (2011). Activated sludge modelling: Development and potential use of a practical applications database. Water Sci. Technol..

[6] Hulsbeek JJW, Kruit J, Roeleveld PJ, Van Loosdrecht MCM (2002). A practical protocol for dynamic modelling of activated sludge systems. Water Sci. Technol..

[7] Langergraber G (2004). A guideline for simulation studies of wastewater treatment plants. Water Sci. Technol..

[8] Melcer H (2004). Methods for Wastewater Characterization in Activated Sludge Modelling.

[9] Vanrolleghem P (2003). A comprehensive model calibration procedure for activated sludge models. Proc. Water Environ. Fed..

[10] Zhu A (2015). A novel protocol for model calibration in biological wastewater treatment. Sci. Rep..

[11] Brun R, Reichert P, Künsch HR (2001). Practical identifiability analysis of large environmental simulation models. Water Res. Res..

[12] Brun R, Kühni M, Siegrist H, Gujer W, Reichert P (2002). Practical identifiability of ASM2d parameters—Systematic selection and tuning of parameter subsets. Water Res..

[13] Gábor A, Villaverde AF, Banga JR (2017). Parameter identifiability analysis and visualization in large-scale kinetic models of biosystems. BMC Syst. Biol..

[14] Noutsopoulos C, Charalambous V, Koumaki E (2020). Evaluating the fate of emerging contaminants in wastewater treatment plants through plant-wide mathematical modelling. Environ. Proc..

[15] Sen D, Mitta P, Randall CW (1994). Performance of fixed film media integrated in activated sludge reactors to enhance nitrogen removal. Water Sci. Technol..

[16] Johnson TL, McQuarrie JP, Shaw AR (2004). Integrated fixed-film activated sludge (IFAS): The new choice for nitrogen removal upgrades in the United States. Proc. Water Environ. Fed..

[17] Di Trapani D, Mannina G, Torregrossa M, Viviani G (2010). Comparison between hybrid moving bed biofilm reactor and activated sludge system: A pilot plant experiment. Water Sci. Technol..

[18] Rahimi Y, Torabian A, Mehrdadi N, Shahmoradi B (2011). Simultaneous nitrification–denitrification and phosphorus removal in a fixed bed sequencing batch reactor (FBSBR). J. Hazard. Mater..

[19] Rosso D (2011). Oxygen transfer and uptake, nutrient removal, and energy footprint of parallel full-scale IFAS and activated sludge processes. Water Res..

[20] Albizuri J, Van Loosdrecht MCM, Larrea L (2009). Extended mixed-culture biofilms (MCB) model to describe integrated fixed film/activated sludge (IFAS) process behaviour. Water Sci. Technol..

[21] Thalla AK, Bhargava R, Kumar P (2010). Nitrification kinetics of activated sludge-biofilm system: A mathematical model. Bioresourc. Technol..

[22] Moretti P (2018). Dynamic modeling of nitrogen removal for a three-stage integrated fixed-film activated sludge process treating municipal wastewater. Bioprocess Biosyst. Eng..

[23] Tao C, Hamouda MA (2019). Steady-state modeling and evaluation of partial nitrification-anammox (PNA) for moving bed biofilm reactor and integrated fixed-film activated sludge processes treating municipal wastewater. J. Water Proc. Eng..

[24] Brockmann, D. *et al*. Model calibration for moving-bed biofilm and integrated fixed-film activated sludge reactors: Experiences with the good biofilm reactor modelling protocol. In *IWA Biofilm Reactors* 10 (IWA Publishing, 2017).

[25] Boltz JP (2012). Framework for biofilm reactor model calibration. WWTmod.

[26] Zeferino JA, Antunes AP, Cunha MC (2009). An efficient simulated annealing algorithm for regional wastewater system planning. Comput.-Aided Civil Infrastruct. Eng..

[27] Cierkens K (2012). Impact of influent data frequency and model structure on the quality of WWTP model calibration and uncertainty. Water Sci. Technol..

[28] Afshar A, Kazemi H, Saadatpour M (2011). Particle swarm optimization for automatic calibration of large scale water quality model (CE-QUAL-W2): Application to Karkheh Reservoir, Iran. Water Res. Manage..

[29] Behrouz MS, Zhu Z, Matott LS, Rabideau AJ (2020). A new tool for automatic calibration of the storm water management model (SWMM). J. Hydrol..

[30] Cho JH, Lee JH (2018). Automatic calibration and performance evaluation of a water quality model for a river greatly influenced by wastewater treatment plant effluent. EPiC Ser. Eng..

[31] Weijers SR, Vanrolleghem PA (1997). A procedure for selecting best identifiable parameters in calibrating activated sludge model no. 1 to full-scale plant data. Water Sci. Technol..

[32] Kim S (2002). Genetic algorithms for the application of activated sludge model no. 1. Water Sci. Technol..

[33] Kim KS, Je CH (2006). Development of a framework of automated water quality parameter optimization and its application. Environ. Geol..

[34] Ye HT (2013). Intelligent estimate method for biological wastewater treatment system model parameters. Comput. Simul..

[35] Du X, Wang J, Jegatheesan V, Shi G (2018). Parameter estimation of activated sludge process based on an improved cuckoo search algorithm. Bioresour. Technol..

[36] Nelder JA, Mead R (1965). A simplex method for function minimization. Comput. J..

[37] Goel L (2020). An extensive review of computational intelligence-based optimization algorithms: Trends and applications. Soft Comput..

[38] Gao F, Han L (2012). Implementing the Nelder-Mead simplex algorithm with adaptive parameters. Comp. Optim. Appl..

[39] Khoja I, Ladhari T, Sakly A, M’sahli F (2018). Parameter identification of an activated sludge wastewater treatment process based on particle swarm optimization method. Math. Prob. Eng..

[40] Meijer SCF, Van Loosdrecht MCM, Heijnen JJ (2001). Metabolic modelling of full-scale biological nitrogen and phosphorus removing WWTP's. Water Res..

[41] Petersen B, Gernaey K, Devisscher M, Dochain D, Vanrolleghem PA (2003). A simplified method to assess structurally identifiable parameters in Monod-based activated sludge models. Water Res..

[42] Zhang XY, Trame MN, Lesko LJ, Schmidt S (2015). Sobol sensitivity analysis: A tool to guide the development and evaluation of systems pharmacology models. CPT Pharmacometrics Syst. Pharmacol..

[43] Link KG (2018). A local and global sensitivity analysis of a mathematical model of coagulation and platelet deposition under flow. PLoS ONE.

[44] Ruano MV, Ribes J, De Pauw DJW, Sin G (2007). Parameter subset selection for the dynamic calibration of activated sludge models (ASMs): Experience versus systems analysis. Water Sci. Technol..

[45] Saltelli A (2008). Global Sensitivity Analysis: The Primer.

[46] Sweetapple C, Guangtao F, Butler D (2013). Identifying key sources of uncertainty in the modelling of greenhouse gas emissions from wastewater treatment. Water Res..

[47] Jolma A, Norton J (2005). Methods of uncertainty treatment in environmental models. Environ. Model. Softw..

[48] Belia E (2009). Wastewater treatment modelling: Dealing with uncertainties. Water Sci. Technol..

[49] Vezzaro L, Mikkelsen PS (2012). Application of global sensitivity analysis and uncertainty quantification in dynamic modelling of micropollutants in stormwater runoff. Environ. Model. Softw..

[50] Singh NK, Kazmi AA, Starkl M (2015). Environmental performance of an integrated fixed-film activated sludge (IFAS) reactor treating actual municipal wastewater during start-up phase. Water Sci. Technol..

[51] Singh NK, Kazmi AA, Starkl M (2016). Treatment performance and microbial diversity under dissolved oxygen stress conditions: Insights from a single stage IFAS reactor treating municipal wastewater. J. Taiw. Inst. Chem. Eng..

[52] Bhatia A, Singh NK, Bhando T, Pathania R, Kazmi AA (2017). Effect of intermittent aeration on microbial diversity in an intermittently aerated IFAS reactor treating municipal wastewater: A field study. J. Environ. Sci. Health A.

[53] Singh NK, Bhatia A, Kazmi AA (2017). Effect of intermittent aeration strategies on treatment performance and microbial community of an IFAS reactor treating municipal waste water. Environ. Technol..

[54] Singh NK, Yadav M, Singh RP, Kazmi AA (2019). Efficacy analysis of a field scale IFAS reactor under different aeration strategies applied at high aeration rates: A statistical comparative analysis for practical feasibility. J. Water Proc. Eng..

[55] Singh RP, Singh NK, Kazmi AA (2020). Environmental sustainability assessment of a fixed media based and package type integrated fixed-film activated sludge reactor in India: A damage-oriented approach. J. Clean. Prod..

[56] Hydromantis, E. S. S. Inc. (2017). GPS-X Technical Reference.

[57] MoEFCC (2015). Standards for Sewage Treatment Plants Along with Time Frame for Implementation, Draft Notification.

[58] Zajac, Z. B. Global sensitivity and uncertainty analysis of spatially distributed watershed models. PhD thesis. University of Florida. ProQuest Dissertations Publishing, 3436446 (2010).

[59] Chu KH, Van Veldhuizen HM, Van Loosdrecht MCM (2003). Respirometric measurement of kinetic parameters: Effect of activated sludge floc size. Water Sci. Technol..

[60] Herman J, Usher W (2017). SALib: An open-source Python library for sensitivity analysis. J. Open Source Soft..

[61] Herman JD, Kollat JB, Reed PM, Wagener T (2013). Method of Morris effectively reduces the computational demands of global sensitivity analysis for distributed watershed models. Hydrol. Earth Syst. Sci..

[62] Morris MD (1991). Factorial sampling plans for preliminary computational experiments. Technology.

[63] Campolongo F, Cariboni J, Saltelli A (2007). An effective screening design for sensitivity analysis of large models. Environ. Model. Softw..

[64] Kucherenko S, Rodriguez-Fernandez M, Pantelides C, Shah N (2009). Monte Carlo evaluation of derivative-based global sensitivity measures. Reliab. Eng. Syst. Saf..

[65] Di Lullo G, Gemechu E, Oni AO, Kumar A (2020). Extending sensitivity analysis using regression to effectively disseminate life cycle assessment results. Int. J. Life Cycl. Assess..

[66] Tian W (2013). A review of sensitivity analysis methods in building energy analysis. Renew. Sustain. Energy Rev..

[67] Zhan CS, Song XM, Xia J, Tong C (2013). An efficient integrated approach for global sensitivity analysis of hydrological model parameters. Environ. Model. Softw..

[68] Brockmann D, Morgenroth E (2007). Comparing global sensitivity analysis for a biofilm model for two-step nitrification using the qualitative screening method of Morris or the quantitative variance-based Fourier Amplitude Sensitivity Test (FAST). Water Sci. Technol..

[69] Sanchez DG, Lacarrière B, Musy M, Bourges B (2014). Application of sensitivity analysis in building energy simulations: Combining first-and second-order elementary effects methods. Energy Build..

[70] Qian G, Mahdi A (2020). Sensitivity analysis methods in the biomedical sciences. Math. Biosci..

[71] Cosenza A, Mannina G, Vanrolleghem PA, Neumann MB (2013). Global sensitivity analysis in wastewater applications: A comprehensive comparison of different methods. Environ. Model. Soft..

[72] Al R, Behera CR, Zubov A, Gernaey KV, Sin G (2019). Meta-modeling based efficient global sensitivity analysis for wastewater treatment plants—An application to the BSM2 model. Comput. Chem. Eng..

[73] Nossent J, Elsen P, Bauwens W (2011). Sobol’sensitivity analysis of a complex environmental model. Environ. Model. Softw..

[74] Ramin E (2014). Influence of selecting secondary settling tank sub-models on the calibration of WWTP models—A global sensitivity analysis using BSM2. Chem. Eng. J..

[75] Hsieh NH, Reisfeld B, Bois FY, Chiu WA (2018). Applying a global sensitivity analysis workflow to improve the computational efficiencies in physiologically-based pharmacokinetic modeling. Front. Pharmacol..

[76] Valverde-Pérez B, Ramin E, Smets BF, Plósz BG (2015). EBP2R—An innovative enhanced biological nutrient recovery activated sludge system to produce growth medium for green microalgae cultivation. Water Res..

[77] Olsson DM, Nelson LS (1975). The Nelder-Mead simplex procedure for function minimization. Technology.

[78] Spendley WGRFR, Hext GR, Himsworth FR (1962). Sequential application of simplex designs in optimisation and evolutionary operation. Technology.

[79] Fan SKS, Liang YC, Zahara E (2006). A genetic algorithm and a particle swarm optimizer hybridized with Nelder-Mead simplex search. Comput. Ind. Eng..

[80] Wright MH (2010). Nelder, Mead, and the other simplex method. Doc. Math..

[81] Magdowski M, Vick R (2010). Estimation of the mathematical parameters of double-exponential pulses using the Nelder-Mead algorithm. IEEE Trans. Electromagn. Compat..

[82] Karahan H (2013). Discussion of “parameter estimation of nonlinear Muskingum models using Nelder-Mead simplex algorithm” by Reza Barati. J. Hydrol. Eng..

[83] Schlesinger S (1979). Terminology for model credibility. SIMULATION.

[84] Ferson S, Oberkampf WL, Ginzburg L (2008). Model validation and predictive capability for the thermal challenge problem. Comput. Method Appl. Mech. Eng..

[85] Sargent RG (2013). Verification and validation of simulation models. J. Simul..

[86] Béline F, Boursier H, Daumer ML, Guiziou F, Paul E (2007). Modelling of biological processes during aerobic treatment of piggery wastewater aiming at process optimisation. Bioresour. Technol..

[87] Farrance I, Frenkel R (2014). Uncertainty in measurement: A review of Monte Carlo simulation using Microsoft Excel for the calculation of uncertainties through functional relationships, including uncertainties in empirically derived constants. Clin. Biochem. Rev..

[88] Isukapalli SS, Georgopoulos PG (2001). Computational Methods for Sensitivity and Uncertainty Analysis for Environmental and Biological Models. EPA/600/R-01–068.

[89] Grady CL, Daigger GT, Love NG, Filipe CD (2011). Biological Wastewater Treatment.

[90] Muller A, Wentzel MC, Loewenthal RE, Ekama GA (2003). Heterotroph anoxic yield in anoxic aerobic activated sludge systems treating municipal wastewater. Water Res..

[91] Ersu CB, Ong SK, Arslankaya E, Lee YW (2010). Impact of solids residence time on biological nutrient removal performance of membrane bioreactor. Water Res..

[92] Nielsen PH, Thomsen TR, Nielsen JL (2004). Bacterial composition of activated sludge-importance for floc and sludge properties. Water Sci. Technol..

[93] Sears K, Alleman JE, Barnard JL, Oleszkiewicz JA (2006). Density and activity characterization of activated sludge flocs. J. Environ. Eng..

[94] Cosenza A, Mannina G, Vanrolleghem PA, Neumann MB (2014). Variance-based sensitivity analysis for wastewater treatment plant modelling. Sci. Total Environ..

[95] Peng Y, Zhu G (2006). Biological nitrogen removal with nitrification and denitrification via nitrite pathway. Appl. Microbiol. Biotechnol..

[96] Liu G, Wang J (2015). Modelling effects of DO and SRT on activated sludge decay and production. Water Res..

[97] Muller AW, Wentzel MC, Ekama GA (2004). Experimental determination of the heterotroph anoxic yield in anoxic-aerobic activated sludge systems treating municipal wastewater. Water SA.

[98] Li B, Qiu Y, Zhang C, Chen L, Shi H (2016). Understanding biofilm diffusion profiles and microbial activities to optimize integrated fixed-film activated sludge process. Chem. Eng. J..

[99] Shaw AR, Johnson TL, Johnson C (2003). Intricacies of modeling the emerging integrated fixed-film activated sludge (IFAS) process. Proc. Water Environ. Fed..

[100] Schraa O, Robinson P, Selegran A (2006). Modeling of an IFAS process with fungal biomass treating pharmaceutical wastewater. Proc. Water Environ. Fed..

[101] Martins AMP, Heijnen JJ, Van Loosdrecht MCM (2003). Effect of dissolved oxygen concentration on sludge settleability. Appl. Microbiol. Biotechnol..

[102] Liao BQ, Lin HJ, Langevin SP, Gao WJ, Leppard GG (2011). Effects of temperature and dissolved oxygen on sludge properties and their role in bioflocculation and settling. Water Res..

[103] Nogaj T (2015). Modeling of organic substrate transformation in the high-rate activated sludge process. Water Sci. Technol..

[104] Wang XJ (2006). Nutrients removal from municipal wastewater by chemical precipitation in a moving bed biofilm reactor. Proc. Biochem..

[105] Chai H (2019). Enhanced simultaneous nitrification and denitrification in treating low carbon-to-nitrogen ratio wastewater: Treatment performance and nitrogen removal pathway. Bioresour. Technol..

[106] Roots P (2019). Comammox nitrospira are the dominant ammonia oxidizers in a mainstream low dissolved oxygen nitrification reactor. Water Res..

[107] Orhon D, Sözen S, Artan N (1996). The effect of heterotrophic yield on the assessment of the correction factor for anoxic growth. Water Sci. Technol..

[108] Spérandio M, Urbain V, Audic JM, Paul E (1999). Use of carbon dioxide evolution rate for determining heterotrophic yield and characterising denitrifying biomass. Water Sci. Technol..

[109] Tas DO (2009). Biodegradability and denitrification potential of settleable chemical oxygen demand in domestic wastewater. Water Environ. Res..

[110] Babuna FG, Orhon D, Çokgör EU, Insel G, Yaprakli B (1998). Modelling of activated sludge for textile wastewaters. Water Sci. Technol..

[111] Ramdani A (2012). Characterization of the heterotrophic biomass and the endogenous residue of activated sludge. Water Res..

[112] Henze M (1999). Activated sludge model no. 2d, ASM2d. Water Sci. Technol..

[113] Van Loosdrecht MC, Henze M (1999). Maintenance, endogeneous respiration, lysis, decay and predation. Water Sci. Technol..

[114] Barker PS, Dold PL (1997). General model for biological nutrient removal activated-sludge systems: Model presentation. Water Environ. Res..

[115] Gujer W, Henze M, Mino T, Van Loosdrecht M (1999). Activated sludge model no. 3. Water Sci. Technol..

[116] Lavallée B, Lessard P, Besser C (2002). Decay rate variability of active heterotrophic biomass. Water Sci. Technol..

[117] Karlikanovaite-Balikci A, Yagci N (2019). Determination and evaluation of kinetic parameters of activated sludge biomass from a sludge reduction system treating real sewage by respirometry testing. J. Environ. Manage..

[118] Henze M (1992). Characterization of wastewater for modelling of activated sludge processes. Water Sci. Technol..

[119] Allı B, İnsel G, Artan N, Orhon D, Sözen S (2018). Behavior of activated sludge systems with an active heterotrophic biomass inflow—A novel perspective for sludge minimization. J. Chem. Technol. Biotechnol..

[120] Çokgör EU, Tas DO, Zengin GE, Insel G (2012). Effect of stabilization on biomass activity. J. Biotechnol..

[121] Sollfrank U, Gujer W (1991). Characterisation of domestic wastewater for mathematical modelling of the activated sludge process. Water Sci. Technol..

[122] Özdemir S, Uçar D, Çokgör EU, Orhon D (2014). Extent of endogenous decay and microbial activity in aerobic stabilization of biological sludge. Desal. Water Treat..

[123] Kappeler J, Gujer W (1992). Estimation of kinetic parameters of heterotrophic biomass under aerobic conditions and characterization of wastewater for activated sludge modelling. Water Sci. Technol..

[124] Insel G, Cokgor E, Tas DO, Sozen S, Orhon D (2015). Impact of the anoxic volume ratio on the dynamics of biological nitrogen removal under extended aeration conditions. Water Air Soil Pollut..

[125] Brockmann, D. *et al*. Model calibration for moving-bed biofilm and integrated fixed-film activated sludge reactors: Experiences with the good biofilm reactor modelling protocol. In *IWA Biofilm Reactors* 10 (IWA Publishing, 2017).

[126] Sen D (2007). Understanding the importance of aerobic mixing, biofilm thickness control and modeling on the success or failure of IFAS systems for biological nutrient removal. Proc. Water Environ. Fed..

[127] Borchert J, Hubbell S, Rupp H (2011). Demonstration of IFAS technology for cold temperature nitrification in lagoon WWTFs at Clare and Ludington, Michigan. Proc. Water Environ. Fed..

[128] Kim HS (2011). Comparison of conventional and integrated fixed-film activated sludge systems: Attached-and suspended-growth functions and quantitative polymerase chain reaction measurements. Water Environ. Res..

