# Supplementary material for: Modelling the performance of an integrated fixed-film activated sludge (IFAS) system: a systematic approach to automated calibration
Source: Sci Rep. 2022 Jun 8;12:9416. doi: 10.1038/s41598-022-13779-w (PMC9177546; doi:10.1038/s41598-022-13779-w)
Supplement: Supplementary file 1 — Supplementary Information. [file 41598_2022_13779_MOESM1_ESM.docx]

**Supplementary Material**

*Table S1. Adjusted input values of the IFAS system within the GPS-X model.*

| **System component** | **Category** | **Section** | **Adjusted parameters** | **Input** | **Unit** |
| --- | --- | --- | --- | --- | --- |
| Influent | Influent | Characterisation | Model | CODstates | - |
|  |  |  | Total COD | 627 | mg/L |
|  |  |  | Total TKN | 44.2 | mg/L |
|  |  |  | Ammonia nitrogen | 33.7 | mg/L |
|  |  |  | VSS/TSS ratio | 0.63 | mgVSS/mgTSS |
|  |  | Flow | Influent flow | 2.9 | m^3^/h |
| IFAS reactor | Model | Model | Model | MANTIS2 | - |
|  |  | Physical | Tanks in series | 1 | - |
|  |  |  | Tank depth | 3.34 | m |
|  |  |  | Volume setup method | Individual | - |
|  |  |  | Individual volumes | 20 | m^3^ |
|  |  |  | Reactor portion filled by media | 0.233 | m^3^/m^3^ |
|  |  |  | Specific surface of media | 385.73 | 1/m |
|  |  |  | water displaced by media | 0.05 | m^3^/m^3^ |
|  |  | Operational | Aeration methoid | Diffused air | - |
|  |  |  | Specific oxygen transfer by… | Using a DO controller | - |
|  |  |  | DO setpoint | 2.5 | mg/L |
|  |  |  | Integral time (controller) | 0.05 | d |
| Settlement tank | Model | Model | Model | SIMPLE1D | - |
|  |  | Physical | Clarifier type | Conical | - |
|  |  |  | Use local temperature | ON | - |
|  |  |  | Local liquid temperature | 30 | ^o^C |
|  |  |  | Conical clarifier input | 6 | m^2^ |
|  |  |  | Water depth at centre | 4 | m^2^ |
|  |  | Operational | Underflow rate (RAS) | 4.8575 | m^3^/h |
|  |  |  | Pumped flow (WAS) | 1.1 | m^3^/d |
| Site properties |  | Plant wide Properties | Liquid temperature | 30 | ^o^C |
|  |  |  | Blower inlet temperature | 30 | ^o^C |
|  |  |  | Elevation above sea level | 372 | m |

*Table S2. Parameters investigated for influence with default values.*

| **Code** | **Parameter name** | **Functional group** | **Default value** | **Unit** |
| --- | --- | --- | --- | --- |
| X1 | Specific adsorption rate | Kinetic heterotrophs | 0.1 | 1/(gCOD/m^3^)/d |
| X2 | saturation/inhibition coefficient for Xs/Xbh | Kinetic heterotrophs | 0.05 | - |
| X3 | Maximum specific growth rate on substrate | Kinetic heterotrophs | 3.2 | 1/d |
| X4 | Saturation/inhibition coefficient for ss | Kinetic heterotrophs | 5 | mgCOD/L |
| X5 | Saturation coefficient for oxygen | Kinetic heterotrophs | 0.2 | mgO^2^/L |
| X6 | Saturation coefficient for nitrogen as nutrient | Kinetic heterotrophs | 0.05 | mgN/L |
| X7 | Switching coefficient for using Nox-N as nutrient | Kinetic heterotrophs | 0.1 | mgN/L |
| X8 | Saturation coefficient for phosphorus (nutrient) | Kinetic heterotrophs | 0.01 | mgP/L |
| X9 | saturation/inhibition cofficient for Sac | Kinetic heterotrophs | 5 | mgCOD/L |
| X10 | Saturation/inhibition coefficient for Spro | Kinetic heterotrophs | 5 | mgCOD/L |
| X11 | Reduction factor for denitrification on nitrate-N | Kinetic heterotrophs | 0.32 | - |
| X12 | Reduction factor for denitrification on nitrite-N | Kinetic heterotrophs | 0.48 | - |
| X13 | Saturation coefficient for nitrite | Kinetic heterotrophs | 0.1 | mgN/L |
| X14 | Saturation coefficient for nitrate | Kinetic heterotrophs | 0.5 | mgN/L |
| X15 | Oxygen inhibition coefficient for denitrification | Kinetic heterotrophs | 0.2 | mgO^2^/L |
| X16 | Aerobic heterotrophic decay rate | Kinetic heterotrophs | 0.62 | 1/d |
| X17 | Anoxic reduction factor for decay rate | Kinetic heterotrophs | 0.9 | - |
| X18 | Anaerobic reduction factor for decay rate | Kinetic heterotrophs | 0.6 | - |
| X19 | Maximum growth rate for ammonia oxidizer | Kinetic AOBs | 0.9 | 1/d |
| X20 | Ammonia saturation coefficient for ammonia oxidizer | Kinetic AOBs | 0.7 | mgN/L |
| X21 | Oxygen saturation for ammonia oxidizer | Kinetic AOBs | 0.25 | mgO2/L |
| X22 | Inihibition coefficient of FA for ammonia oxidizer | Kinetic AOBs | 50 | mgN/L |
| X23 | Inhibition coefficient of FNA for ammonia oxidizer | Kinetic AOBs | 0.2 | mgN/L |
| X24 | Ammonia oxidizer aerobic decay rate | Kinetic AOBs | 0.17 | 1/d |
| X25 | Anoxic reduction factor for decay rate | Kinetic AOBs | 0.5 | - |
| X26 | Anaerobic reduction factor decay rate | Kinetic AOBs | 0.3 | - |
| X27 | Maximum growth rate for nitrite oxidizer | Kinetic NOBs | 1 | 1/d |
| X28 | Nitrite saturation coefficient for nitrite oxidizer | Kinetic NOBs | 0.5 | mgN/L |
| X29 | Oxygen saturation for nitrite oxidizer | Kinetic NOBs | 0.68 | mgO2/L |
| X30 | Inhibition coefficient of FA for nitrite oxidizer | Kinetic NOBs | 1 | mgN/L |
| X31 | Inhibition coefficient of FNA for nitrite oxidizer | Kinetic NOBs | 0.09 | mgN/L |
| X32 | Nitrite oxidizer decay rate | Kinetic NOBs | 0.17 | 1/d |
| X33 | Anoxic reduction factor for decay rate | Kinetic NOBs | 0.5 | - |
| X34 | Anaerobic reduction factor for decay rate | Kinetic NOBs | 0.3 | - |
| X35 | Maximum growth rate of anammox bacteria | Kinetic Anammox | 0.0186 | 1/d |
| X36 | Ammonia saturation for anammox bacteria | Kinetic Anammox | 0.73 | mgN/L |
| X37 | Nitrite saturation coefficient for anammox bacteria | Kinetic Anammox | 0.5 | mgN/L |
| X38 | Oxygen saturation/inhibition for anammox bacteria | Kinetic Anammox | 0.1 | mgO2/L |
| X39 | Aerobic decay rate of anammox bacteria | Kinetic Anammox | 0.0058 | 1/d |
| X40 | Anoxic reduction factor for decay rate | Kinetic Anammox | 0.5 | - |
| X41 | Anaerobic reduction factor for decay rate | Kinetic Anammox | 0.3 | - |
| X42 | Hydrolysis rate constant for xs | Kinetic hydrolosis | 3 | 1/d |
| X43 | Saturation coefficient for particulate COD | Kinetic hydrolosis | 0.1 | - |
| X44 | Anoxic hydrolysis reduction factor | Kinetic hydrolosis | 0.8 | - |
| X45 | Anaerobic hydrolysis reduction factor | Kinetic hydrolosis | 0.4 | - |
| X46 | Saturation/inhibition coefficient for Nox | Kinetic hydrolosis | 0.5 | mgN/L |
| X47 | Hydrolysis rate constant for inert residue | Kinetic hydrolosis | 0.03 | 1/d |
| X48 | Saturation coefficient for inert residue | Kinetic hydrolosis | 1 | - |
| X49 | Hydrolysis rate constant inert organics | Kinetic hydrolosis | 0.03 | 1/d |
| X50 | Saturation coefficient for inert organics | Kinetic hydrolosis | 1 | - |
| X51 | Ammonification rate | Kinetic ammonification | 0.08 | m^3^/gCOD/d |
| X52 | Aerobic heterotrophic yield on soluble substrate (50%) | Stoichiometric | 0.666 | gCOD/gCOD |
| X53 | Anoxic heterotrophic yield on soluble substrate (50%) | Stoichiometric | 0.533 | gCOD/gCOD |
| X54 | Ammonia-oxidizer yield | Stoichiometric | 0.18 | gCOD/gN |
| X55 | Nitrite-oxidizer yield | Stoichiometric | 0.06 | gCOD/gN |
| X56 | Biomass yield on NH4-N | Stoichiometric | 0.1675 | gCOD/gN |
| X57 | Aerobic yield on PAO growth | Stoichiometric | 0.639 | gCOD/gCOD |
| X58 | Anoxic yield on PAO growth | Stoichiometric | 0.511 | gCOD/gCOD |
| X59 | PHA storage yield | Stoichiometric | 0.4 | gP/gCOD |
| X60 | Xpp storage yield | Stoichiometric | 0.2 | gP/gCOD |
| X61 | Unbiodegradable fraction from cell decay | Stoichiometric | 0.08 | gCOD/gCOD |
| X62 | Sludge volume index | Settling factors | 150 | mL/g |
| X63 | Clarification (0-bad,1-good) | Settling factors | 0.5 | - |
| X64 | Maximum settling velocity | Settling factors | 274 | m/d |
| X65 | Ratio of compression to hindered settling parameter | Settling factors | 2 | - |
| X66 | Critical concentration for compression settling | Settling factors | 8000 | mg/L |
| X67 | Non-settleable fraction | Settling factors | 0.001 | - |
| X68 | Quiescent zone maximum upflow velocity | Settling factors | 100 | m/d |

*
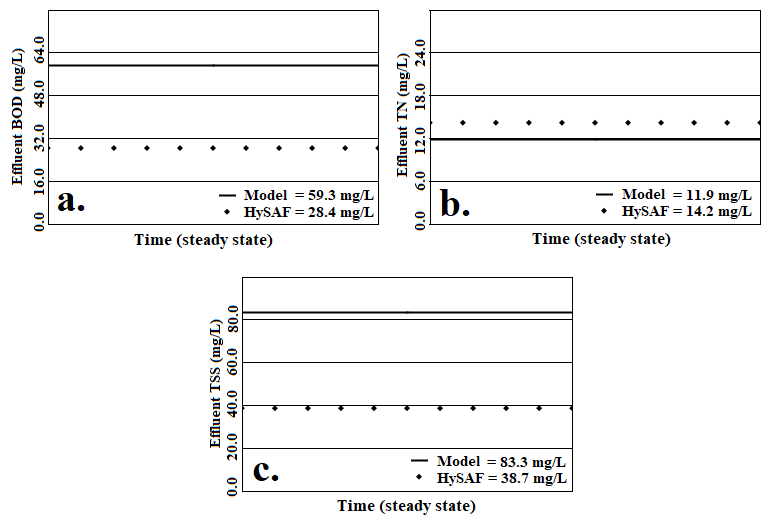
*

*Figure S1. Pre-calibration accuracy of model outputs to observed outputs.*

**Python script S1. Sensitivity Analysis – Method of Morris**

**import** **pandas** **as** **pd**

**import** **numpy** **as** **np**

**import** **sys**

**import** **matplotlib.pyplot** **as** **plt**

**from** **SALib.sample** **import** saltelli

**from** **SALib.sample.morris** **import** sample

**from** **SALib.analyze** **import** morris

**from** **SALib.plotting.morris** **import** horizontal_bar_plot, covariance_plot, \

sample_histograms

**from** **threading** **import** Thread

**import** **functools**

**import** **time**

**import** **traceback**

timeout_min = **15**

**def** **timeout**(timeout):

**def** **deco**(func):

**@functools.wraps**(func)

**def** **wrapper**(*args, **kwargs):

res = [**Exception**('function [%s] timeout [%s seconds] exceeded!' % (func.__name__, timeout))]

**def** **newFunc**():

**try**:

res[**0**] = func(*args, **kwargs)

**except** **Exception** **as** e:

res[**0**] = e

t = Thread(target=newFunc)

t.daemon = True

**try**:

t.start()

t.join(timeout)

**except** **Exception** **as** je:

**print**('error starting thread')

**raise** je

ret = res[**0**]

**if** isinstance(ret, **BaseException**):

**raise** ret

**return** ret

**return** wrapper

**return** deco

##### SENSITIVITY ANALYSIS - Generate sample phase #####

# Define problem

problem = {

'num_vars': **68**,

'names': ['X1', 'X2', 'X3', 'X4', 'X5', 'X6', 'X7', 'X8', 'X9', 'X10',

'X11', 'X12', 'X13', 'X14', 'X15', 'X16', 'X17', 'X18', 'X19', 'X20',

'X21', 'X22', 'X23', 'X24', 'X25', 'X26', 'X27', 'X28', 'X29', 'X30',

'X31', 'X32', 'X33', 'X34', 'X35', 'X36', 'X37', 'X38', 'X39', 'X40',

'X41', 'X42', 'X43', 'X44', 'X45', 'X46', 'X47', 'X48', 'X49', 'X50',

'X51', 'X52', 'X53', 'X54', 'X55', 'X56', 'X57', 'X58', 'X59', 'X60',

'X61', 'X62', 'X63', 'X64', 'X65', 'X66', 'X67', 'X68'],

'bounds': [[**0.05**, **0.15**],

[**0.025**, **0.075**],

[**1.6**, **4.8**],

[**2.5**, **7.5**],

[**0.1**, **0.3**],

[**0.025**, **0.075**],

[**0.05**, **0.15**],

[**0.005**, **0.015**],

[**2.5**, **7.5**],

[**2.5**, **7.5**],

[**0.16**, **0.48**],

[**0.24**, **0.72**],

[**0.05**, **0.15**],

[**0.25**, **0.75**],

[**0.1**, **0.3**],

[**0.31**, **0.93**],

[**0.45**, **1.35**],

[**0.3**, **0.9**],

[**0.45**, **1.35**],

[**0.35**, **1.05**],

[**0.125**, **0.375**],

[**25**, **75**],

[**0.1**, **0.3**],

[**0.085**, **0.255**],

[**0.25**, **0.75**],

[**0.15**, **0.45**],

[**0.5**, **1.5**],

[**0.25**, **0.75**],

[**0.34**, **1.02**],

[**0.5**, **1.5**],

[**0.045**, **0.135**],

[**0.085**, **0.255**],

[**0.25**, **0.75**],

[**0.15**, **0.45**],

[**0.009275**, **0.027825**],

[**0.365**, **1.095**],

[**0.25**, **0.75**],

[**0.05**, **0.15**],

[**0.0029**, **0.0087**],

[**0.25**, **0.75**],

[**0.15**, **0.45**],

[**1.5**, **4.5**],

[**0.05**, **0.15**],

[**0.4**, **1.2**],

[**0.2**, **0.6**],

[**0.25**, **0.75**],

[**0.015**, **0.045**],

[**0.5**, **1.5**],

[**0.015**, **0.045**],

[**0.5**, **1.5**],

[**0.04**, **0.12**],

[**0.333**, **0.999**],

[**0.2665**, **0.7995**],

[**0.09**, **0.27**],

[**0.03**, **0.09**],

[**0.08375**, **0.25125**],

[**0.3195**, **0.9585**],

[**0.2555**, **0.7665**],

[**0.2**, **0.6**],

[**0.1**, **0.3**],

[**0.04**, **0.12**],

[**75**, **225**],

[**0.25**, **0.75**],

[**137**, **411**],

[**1**, **3**],

[**4000**, **12000**],

[**0.0005**, **0.0015**],

[**50**, **150**]]

}

# Generate the sample in a dataframe that will form the input values

InputMoM = sample(problem, **100**, num_levels=**4**)

# Create header row with cryptic names for GPS-X to use and name df appropriately

MoM_input100 = pd.DataFrame(InputMoM,

columns=['qadsconwwstream', 'kadsconwwstream', 'muhconwwstream', 'khssconwwstream',

'khsoconwwstream', 'khsnhconwwstream', 'kIhsnhconwwstream', 'khspconwwstream', 'khsacconwwstream',

'khsproconwwstream', 'no3grohetconwwstream', 'no2grohetconwwstream', 'khsno2conwwstream', 'khsno3conwwstream',

'khisono3conwwstream', 'bhconwwstream', 'nanoxxbhconwwstream', 'nanaerxbhconwwstream',

'munhconwwstream', 'ka1snhconwwstream', 'ka1soconwwstream', 'kifsnh3xbaiconwwstream',

'kifsno2xbaiconwwstream', 'bnhconwwstream', 'nanoxxbaiconwwstream', 'nanaerxbaiconwwstream',

'muno2conwwstream', 'ka2sno2conwwstream', 'ka2soconwwstream', 'kifsnh3xbaaconwwstream',

'kifsno2xbaaconwwstream', 'bno2conwwstream', 'nanoxxbaaconwwstream', 'nanaerxbaaconwwstream',

'muaxconwwstream', 'kaxsnhconwwstream', 'kaxsno2conwwstream', 'kaxsoconwwstream', 'baxconwwstream',

'nanoxxbaxconwwstream', 'nanaerxbaxconwwstream', 'khconwwstream', 'khxsconwwstream', 'nsanoxconwwstream',

'nsanaerconwwstream', 'khsnoxconwwstream', 'kbxuconwwstream', 'khxuconwwstream', 'kbxiconwwstream',

'khxiconwwstream', 'kammoconwwstream', 'yhairconwwstream', 'yhanoconwwstream', 'ya1conwwstream',

'ya2conwwstream', 'yaxconwwstream', 'ypairconwwstream', 'ypanoconwwstream', 'ypo4conwwstream',

'yppphaconwwstream', 'fuconwwstream', 'sviweff', 'clarifweff', 'vbndweff', 'rcomptohindweff',

'xcriticalweff', 'fnsweff', 'vumiweff'])

# Show df to confirm headers attached properly

**print**(MoM_input100)

sys.stdout.flush()

# Save input df as an excel file for record

# Write the new dataframe with the generated input values and cryptic headers to an excel file

# Create a Pandas Excel writer object using xlsxwriter as the engine.

writer = pd.ExcelWriter('MoM_input100.xlsx',

engine='xlsxwriter')

# write the dataframe to the worksheet

MoM_input100.to_excel(writer, sheet_name='MoM Input Values')

# close the pandas excel writer object and output the excel file

writer.save()

##### GPS-X Part #####

current_row = **0**

simutation = **0**

# list to keep output values

BOD_outputs = []

TN_outputs = []

TSS_outputs = []

StopTime = **0.0**

# runs when new simulation started

**def** **start**():

**global** current_row, StopTime

# set new variable values using dataframe row by index

gpsx.setTstop(StopTime)

gpsx.setValue('qadsconwwstream', MoM_input100.iloc[current_row]['qadsconwwstream'])

gpsx.setValue('kadsconwwstream', MoM_input100.iloc[current_row]['kadsconwwstream'])

gpsx.setValue('muhconwwstream', MoM_input100.iloc[current_row]['muhconwwstream'])

gpsx.setValue('khssconwwstream', MoM_input100.iloc[current_row]['khssconwwstream'])

gpsx.setValue('khsoconwwstream', MoM_input100.iloc[current_row]['khsoconwwstream'])

gpsx.setValue('khsnhconwwstream', MoM_input100.iloc[current_row]['khsnhconwwstream'])

gpsx.setValue('kIhsnhconwwstream', MoM_input100.iloc[current_row]['kIhsnhconwwstream'])

gpsx.setValue('khspconwwstream', MoM_input100.iloc[current_row]['khspconwwstream'])

gpsx.setValue('khsacconwwstream', MoM_input100.iloc[current_row]['khsacconwwstream'])

gpsx.setValue('khsproconwwstream', MoM_input100.iloc[current_row]['khsproconwwstream'])

gpsx.setValue('no3grohetconwwstream', MoM_input100.iloc[current_row]['no3grohetconwwstream'])

gpsx.setValue('no2grohetconwwstream', MoM_input100.iloc[current_row]['no2grohetconwwstream'])

gpsx.setValue('khsno2conwwstream', MoM_input100.iloc[current_row]['khsno2conwwstream'])

gpsx.setValue('khsno3conwwstream', MoM_input100.iloc[current_row]['khsno3conwwstream'])

gpsx.setValue('khisono3conwwstream', MoM_input100.iloc[current_row]['khisono3conwwstream'])

gpsx.setValue('bhconwwstream', MoM_input100.iloc[current_row]['bhconwwstream'])

gpsx.setValue('nanoxxbhconwwstream', MoM_input100.iloc[current_row]['nanoxxbhconwwstream'])

gpsx.setValue('nanaerxbhconwwstream', MoM_input100.iloc[current_row]['nanaerxbhconwwstream'])

gpsx.setValue('munhconwwstream', MoM_input100.iloc[current_row]['munhconwwstream'])

gpsx.setValue('ka1snhconwwstream', MoM_input100.iloc[current_row]['ka1snhconwwstream'])

gpsx.setValue('ka1soconwwstream', MoM_input100.iloc[current_row]['ka1soconwwstream'])

gpsx.setValue('kifsnh3xbaiconwwstream', MoM_input100.iloc[current_row]['kifsnh3xbaiconwwstream'])

gpsx.setValue('kifsno2xbaiconwwstream', MoM_input100.iloc[current_row]['kifsno2xbaiconwwstream'])

gpsx.setValue('bnhconwwstream', MoM_input100.iloc[current_row]['bnhconwwstream'])

gpsx.setValue('nanoxxbaiconwwstream', MoM_input100.iloc[current_row]['nanoxxbaiconwwstream'])

gpsx.setValue('nanaerxbaiconwwstream', MoM_input100.iloc[current_row]['nanaerxbaiconwwstream'])

gpsx.setValue('muno2conwwstream', MoM_input100.iloc[current_row]['muno2conwwstream'])

gpsx.setValue('ka2sno2conwwstream', MoM_input100.iloc[current_row]['ka2sno2conwwstream'])

gpsx.setValue('ka2soconwwstream', MoM_input100.iloc[current_row]['ka2soconwwstream'])

gpsx.setValue('kifsnh3xbaaconwwstream', MoM_input100.iloc[current_row]['kifsnh3xbaaconwwstream'])

gpsx.setValue('kifsno2xbaaconwwstream', MoM_input100.iloc[current_row]['kifsno2xbaaconwwstream'])

gpsx.setValue('bno2conwwstream', MoM_input100.iloc[current_row]['bno2conwwstream'])

gpsx.setValue('nanoxxbaaconwwstream', MoM_input100.iloc[current_row]['nanoxxbaaconwwstream'])

gpsx.setValue('nanaerxbaaconwwstream', MoM_input100.iloc[current_row]['nanaerxbaaconwwstream'])

gpsx.setValue('muaxconwwstream', MoM_input100.iloc[current_row]['muaxconwwstream'])

gpsx.setValue('kaxsnhconwwstream', MoM_input100.iloc[current_row]['kaxsnhconwwstream'])

gpsx.setValue('kaxsno2conwwstream', MoM_input100.iloc[current_row]['kaxsno2conwwstream'])

gpsx.setValue('kaxsoconwwstream', MoM_input100.iloc[current_row]['kaxsoconwwstream'])

gpsx.setValue('baxconwwstream', MoM_input100.iloc[current_row]['baxconwwstream'])

gpsx.setValue('nanoxxbaxconwwstream', MoM_input100.iloc[current_row]['nanoxxbaxconwwstream'])

gpsx.setValue('nanaerxbaxconwwstream', MoM_input100.iloc[current_row]['nanaerxbaxconwwstream'])

gpsx.setValue('khconwwstream', MoM_input100.iloc[current_row]['khconwwstream'])

gpsx.setValue('khxsconwwstream', MoM_input100.iloc[current_row]['khxsconwwstream'])

gpsx.setValue('nsanoxconwwstream', MoM_input100.iloc[current_row]['nsanoxconwwstream'])

gpsx.setValue('nsanaerconwwstream', MoM_input100.iloc[current_row]['nsanaerconwwstream'])

gpsx.setValue('khsnoxconwwstream', MoM_input100.iloc[current_row]['khsnoxconwwstream'])

gpsx.setValue('kbxuconwwstream', MoM_input100.iloc[current_row]['kbxuconwwstream'])

gpsx.setValue('khxuconwwstream', MoM_input100.iloc[current_row]['khxuconwwstream'])

gpsx.setValue('kbxiconwwstream', MoM_input100.iloc[current_row]['kbxiconwwstream'])

gpsx.setValue('khxiconwwstream', MoM_input100.iloc[current_row]['khxiconwwstream'])

gpsx.setValue('kammoconwwstream', MoM_input100.iloc[current_row]['kammoconwwstream'])

gpsx.setValue('yhairconwwstream', MoM_input100.iloc[current_row]['yhairconwwstream'])

gpsx.setValue('yhanoconwwstream', MoM_input100.iloc[current_row]['yhanoconwwstream'])

gpsx.setValue('ya1conwwstream', MoM_input100.iloc[current_row]['ya1conwwstream'])

gpsx.setValue('ya2conwwstream', MoM_input100.iloc[current_row]['ya2conwwstream'])

gpsx.setValue('yaxconwwstream', MoM_input100.iloc[current_row]['yaxconwwstream'])

gpsx.setValue('ypairconwwstream', MoM_input100.iloc[current_row]['ypairconwwstream'])

gpsx.setValue('ypanoconwwstream', MoM_input100.iloc[current_row]['ypanoconwwstream'])

gpsx.setValue('ypo4conwwstream', MoM_input100.iloc[current_row]['ypo4conwwstream'])

gpsx.setValue('yppphaconwwstream', MoM_input100.iloc[current_row]['yppphaconwwstream'])

gpsx.setValue('fuconwwstream', MoM_input100.iloc[current_row]['fuconwwstream'])

gpsx.setValue('sviweff', MoM_input100.iloc[current_row]['sviweff'])

gpsx.setValue('clarifweff', MoM_input100.iloc[current_row]['clarifweff'])

gpsx.setValue('vbndweff', MoM_input100.iloc[current_row]['vbndweff'])

gpsx.setValue('rcomptohindweff', MoM_input100.iloc[current_row]['rcomptohindweff'])

gpsx.setValue('xcriticalweff', MoM_input100.iloc[current_row]['xcriticalweff'])

gpsx.setValue('fnsweff', MoM_input100.iloc[current_row]['fnsweff'])

gpsx.setValue('vumiweff', MoM_input100.iloc[current_row]['vumiweff'])

# eor() function executed once at end of simulation

# finished set True is required to terminate the runSim() function

#

**def** **eor**():

# add bodweff final value to the list of BOD-outputs when simulation ended

**try**:

BOD_outputs.append(gpsx.getValue('bodweff'))

TN_outputs.append(gpsx.getValue('tnweff'))

TSS_outputs.append(gpsx.getValue('xweff'))

**except** **Exception** **as** e:

# add info about exception to the result

BOD_outputs.append('Simulation failed: {}'.format(str(e)))

TN_outputs.append('Simulation failed: {}'.format(str(e)))

TSS_outputs.append('Simulation failed: {}'.format(str(e)))

**global** finished

finished = True

# cint() function executed at every communication interval

#

**def** **cint**():

# just print current BOD value

**print**(gpsx.getValue('bodweff'))

**print**(gpsx.getValue('tnweff'))

**print**(gpsx.getValue('xweff'))

sys.stdout.flush()

**pass**

**@timeout**(**60***timeout_min)

**def** **start_iteration**():

gpsx.resetSim()

runSim()

# run simulation for each row in dataset

**for** i **in** range(len(MoM_input100)):

# get index of row and make it global

current_row = i

**try**:

simutation+=**1**

**print**("Current simulation: {}/{}".format(str(simutation), str(len(MoM_input100))))

sys.stdout.flush()

# run simulatioion itself

# reset simultaion

**while** True:

**try**:

start_iteration()

**break**

**except** **Exception** **as** e:

**print**(str(e))

**print**("Timeout reached! Trying again!")

sys.stdout.flush()

**continue**

**except** **Exception**:

**pass**

# create new column in dataframe - 'Effluent BOD'

MoM_input100['Effluent BOD'] = BOD_outputs

MoM_input100['Effluent TN'] = TN_outputs

MoM_input100['Effluent TSS'] = TSS_outputs

writer = pd.ExcelWriter('MoM_output100.xlsx',

engine='xlsxwriter')

**print**(BOD_outputs)

**print**(TN_outputs)

**print**(TSS_outputs)

**print**(len(BOD_outputs))

**print**(len(TN_outputs))

**print**(len(TSS_outputs))

sys.stdout.flush()

# write the dataframe to the worksheet

MoM_input100.to_excel(writer, sheet_name='MoM Input Output Values')

writer.save()

# This is calling the input and output data from the model or X and Y

data = pd.read_excel(r'MoM_output100.xlsx')

X = pd.DataFrame(data,

columns=['qadsconwwstream', 'kadsconwwstream', 'muhconwwstream', 'khssconwwstream',

'khsoconwwstream', 'khsnhconwwstream', 'kIhsnhconwwstream', 'khspconwwstream', 'khsacconwwstream',

'khsproconwwstream', 'no3grohetconwwstream', 'no2grohetconwwstream', 'khsno2conwwstream', 'khsno3conwwstream',

'khisono3conwwstream', 'bhconwwstream', 'nanoxxbhconwwstream', 'nanaerxbhconwwstream',

'munhconwwstream', 'ka1snhconwwstream', 'ka1soconwwstream', 'kifsnh3xbaiconwwstream',

'kifsno2xbaiconwwstream', 'bnhconwwstream', 'nanoxxbaiconwwstream', 'nanaerxbaiconwwstream',

'muno2conwwstream', 'ka2sno2conwwstream', 'ka2soconwwstream', 'kifsnh3xbaaconwwstream',

'kifsno2xbaaconwwstream', 'bno2conwwstream', 'nanoxxbaaconwwstream', 'nanaerxbaaconwwstream',

'muaxconwwstream', 'kaxsnhconwwstream', 'kaxsno2conwwstream', 'kaxsoconwwstream', 'baxconwwstream',

'nanoxxbaxconwwstream', 'nanaerxbaxconwwstream', 'khconwwstream', 'khxsconwwstream', 'nsanoxconwwstream',

'nsanaerconwwstream', 'khsnoxconwwstream', 'kbxuconwwstream', 'khxuconwwstream', 'kbxiconwwstream',

'khxiconwwstream', 'kammoconwwstream', 'yhairconwwstream', 'yhanoconwwstream', 'ya1conwwstream',

'ya2conwwstream', 'yaxconwwstream', 'ypairconwwstream', 'ypanoconwwstream', 'ypo4conwwstream',

'yppphaconwwstream', 'fuconwwstream', 'sviweff', 'clarifweff', 'vbndweff', 'rcomptohindweff',

'xcriticalweff', 'fnsweff', 'vumiweff'])

**try**:

Y_BOD = pd.DataFrame(data, columns=['Effluent BOD'])

Si_BOD = morris.analyze(problem, X.to_numpy(), Y_BOD.to_numpy(), conf_level=**0.95**,

print_to_console=True, num_levels=**4**)

Y_TN = pd.DataFrame(data, columns=['Effluent TN'])

Si_TN = morris.analyze(problem, X.to_numpy(), Y_TN.to_numpy(), conf_level=**0.95**,

print_to_console=True, num_levels=**4**)

Y_TSS = pd.DataFrame(data, columns=['Effluent TSS'])

Si_TSS = morris.analyze(problem, X.to_numpy(), Y_TSS.to_numpy(), conf_level=**0.95**,

print_to_console=True, num_levels=**4**)

# Then to graphically represent the results

# Plot horizontal bar plots showing results

# First describe the layout of the plots, in this case 2 side by side

**print**(plt.subplots(**3**, **2**))

fig, ((ax1, ax2), (ax3, ax4), (ax5, ax6)) = plt.subplots(**3**, **2**)

# Next define the first plot, the horizontal bar plot showing the Mu_star of each parameter

# Mu = the absolute of the mean elementary effect = the overall influence of parameter on model output

horizontal_bar_plot(ax1, Si_BOD, {}, sortby='mu_star')

# Next define the second plot which shows Sigma vs Mu_Star

# Sigma = the standard deviation of the elementary effect = Describes non-linear effects and interactions

covariance_plot(ax2, Si_BOD, {})

horizontal_bar_plot(ax3, Si_TN, {}, sortby='mu_star')

covariance_plot(ax4, Si_TN, {})

horizontal_bar_plot(ax5, Si_TSS, {}, sortby='mu_star')

covariance_plot(ax6, Si_TSS, {})

plt.show()

**except** **Exception**:

**print**(traceback.format_exc())

exit()

**Python script S2. Sensitivity Analysis – Sobol analysis (example BOD)**

**import** **pandas** **as** **pd**

**import** **numpy** **as** **np**

**import** **seaborn** **as** **sns**

**import** **sys**

**import** **matplotlib.pyplot** **as** **plt**

**from** **SALib.sample** **import** saltelli

**from** **SALib.analyze** **import** sobol

**from** **threading** **import** Thread

**import** **functools**

**import** **time**

timeout_min = **15**

**def** **timeout**(timeout):

**def** **deco**(func):

**@functools.wraps**(func)

**def** **wrapper**(*args, **kwargs):

res = [**Exception**('function [%s] timeout [%s seconds] exceeded!' % (func.__name__, timeout))]

**def** **newFunc**():

**try**:

res[**0**] = func(*args, **kwargs)

**except** **Exception** **as** e:

res[**0**] = e

t = Thread(target=newFunc)

t.daemon = True

**try**:

t.start()

t.join(timeout)

**except** Exceptiona **as** je:

**print**('error starting thread')

**raise** je

ret = res[**0**]

**if** isinstance(ret, **BaseException**):

**raise** ret

**return** ret

**return** wrapper

**return** deco

##### SENSITIVITY ANALYSIS - Generate sample phase #####

# Define problem

# Parameters are deviated 50 - 150% for the GSA as utilized in previous studies (Link et al. 2018)

problem = {

'num_vars': **10**,

'names': ['X52', 'X16', 'X53', 'X42', 'X19', 'X7', 'X15', 'X24', 'X20', 'X3'],

'bounds': [[**0.333**, **0.999**],

[**0.31**, **0.93**],

[**0.2665**, **0.7995**],

[**1.5**, **4.5**],

[**0.45**, **1.35**],

[**0.05**, **0.15**],

[**0.1**, **0.3**],

[**0.085**, **0.255**],

[**0.35**, **1.05**],

[**1.6**, **4.8**]]

}

# Generate the sample in a dataframe that will form the input values

InputBOD_sobol = saltelli.sample(problem, **10000**)

# Create header row with cryptic names for GPS-X to use and name df appropriately

InputBOD_sobol.shape

BOD_sobol_input = pd.DataFrame(InputBOD_sobol,

columns=['yhairconwwstream', 'bhconwwstream', 'yhanoconwwstream',

'khconwwstream', 'munhconwwstream', 'klhsnhconwwstream',

'khisono3conwwstream', 'bnhconwwstream','ka1snhconwwstream',

'muhconwwstream'])

# Show df to confirm headers attached properly

**print**(BOD_sobol_input)

sys.stdout.flush()

# Save input df as an excel file for record

# Write the new dataframe with the generated input values and cryptic headers to an excel file

# Create a Pandas Excel writer object using xlsxwriter as the engine.

writer = pd.ExcelWriter('BOD_sobol_input.xlsx',

engine='xlsxwriter')

# write the dataframe to the worksheet

BOD_sobol_input.to_excel(writer, sheet_name='BOD Input Values')

# close the pandas excel writer object and output the excel file

writer.save()

##### GPS-X Part #####

current_row = **0**

simutation = **0**

# list to keep output values

BOD_outputs = []

StopTime = **0.0**

# runs when new simulation started

**def** **start**():

**global** current_row, StopTime

gpsx.setTstop(StopTime)

gpsx.setValue('yhairconwwstream', BOD_sobol_input.iloc[current_row]['yhairconwwstream'])

gpsx.setValue('bhconwwstream', BOD_sobol_input.iloc[current_row]['bhconwwstream'])

gpsx.setValue('yhanoconwwstream', BOD_sobol_input.iloc[current_row]['yhanoconwwstream'])

gpsx.setValue('khconwwstream', BOD_sobol_input.iloc[current_row]['khconwwstream'])

gpsx.setValue('munhconwwstream', BOD_sobol_input.iloc[current_row]['munhconwwstream'])

gpsx.setValue('klhsnhconwwstream', BOD_sobol_input.iloc[current_row]['klhsnhconwwstream'])

gpsx.setValue('khisono3conwwstream', BOD_sobol_input.iloc[current_row]['khisono3conwwstream'])

gpsx.setValue('bnhconwwstream', BOD_sobol_input.iloc[current_row]['bnhconwwstream'])

gpsx.setValue('ka1snhconwwstream', BOD_sobol_input.iloc[current_row]['ka1snhconwwstream'])

gpsx.setValue('muhconwwstream', BOD_sobol_input.iloc[current_row]['muhconwwstream'])

# eor() function executed once at end of simulation

# finished set True is required to terminate the runSim() function

#

**def** **eor**():

# add bodweff final value to the list of BOD-outputs when simulation ended

**try**:

BOD_outputs.append(gpsx.getValue('bodweff'))

**except** **Exception** **as** e:

# add info about exception to the result

BOD_outputs.append('Simulation failed: {}'.format(str(e)))

**global** finished

finished = True

# cint() function executed at every communication interval

#

**def** **cint**():

# just print current BOD value

**print**(gpsx.getValue('bodweff'))

sys.stdout.flush()

**pass**

**@timeout**(**60***timeout_min)

**def** **start_iteration**():

gpsx.resetSim()

runSim()

# run simulation for each row in dataset

**for** i **in** range(len(BOD_sobol_input)):

# get index of row and make it global

current_row = i

**try**:

simutation+=**1**

**print**("Current simulation: {}/{}".format(str(simutation), str(len(BOD_sobol_input))))

sys.stdout.flush()

# run simulation itself

# reset simulation

**while** True:

**try**:

start_iteration()

**break**

**except** **Exception**:

**print**("Timeout reached! Trying again!")

sys.stdout.flush()

**continue**

**except** **Exception**:

**pass**

**print**(BOD_outputs)

# create new column in dataframe - 'Effluent BOD'

BOD_sobol_input['Effluent BOD'] = BOD_outputs

writer = pd.ExcelWriter('BOD_sobol_output.xlsx',

engine='xlsxwriter')

sys.stdout.flush()

BOD_sobol_input.to_excel(writer, sheet_name='BOD Input Output Values')

writer.save()

# To visualise S1 and ST as table

Si_filter = {k:Si[k] **for** k **in** ['ST','ST_conf','S1','S1_conf']}

Si_df = pd.DataFrame(Si_filter, index=problem['names'])

Si_df

# To visualise S1 and ST as bar chart with error bars

fig, ax = plt.subplots(**1**)

indices = Si_df[['S1','ST']]

err = Si_df[['S1_conf','ST_conf']]

indices.plot.bar(yerr=err.values.T,ax=ax)

fig.set_size_inches(**8**,**4**)

plt.axhline(**0**, linestyle='-', linewidth=**1**, color='k')

plt.xlabel("Parameters", fontsize=**13**)

plt.ylabel("Sobol indicie", fontsize=**13**)

plt.show()

# To visualise S2 effects

**def** **normalize**(x, xmin, xmax):

**return** (x-xmin)/(xmax-xmin)

**def** **plot_circles**(ax, locs, names, max_s, stats, smax, smin, fc, ec, lw,

zorder):

s = np.asarray([stats[name] **for** name **in** names])

s = **0.01** + max_s * np.sqrt(normalize(s, smin, smax))

fill = True

**for** loc, name, si **in** zip(locs, names, s):

**if** fc=='w':

fill=False

**else**:

ec='none'

x = np.cos(loc)

y = np.sin(loc)

circle = plt.Circle((x,y), radius=si, ec=ec, fc=fc, transform=ax.transData._b,

zorder=zorder, lw=lw, fill=True)

ax.add_artist(circle)

**def** **filter**(sobol_indices, names, locs, criterion, threshold):

**if** criterion **in** ['ST', 'S1', 'S2']:

data = sobol_indices[criterion]

data = np.abs(data)

data = data.flatten() # flatten in case of S2

# TODO:: remove nans

filtered = ([(name, locs[i]) **for** i, name **in** enumerate(names) **if**

data[i]>threshold])

filtered_names, filtered_locs = zip(*filtered)

**elif** criterion **in** ['ST_conf', 'S1_conf', 'S2_conf']:

**raise** **NotImplementedError**

**else**:

**raise** **ValueError**('unknown value for criterion')

**return** filtered_names, filtered_locs

**def** **plot_sobol_indices**(sobol_indices, criterion='ST', threshold=**0.01**):

max_linewidth_s2 = **15**#25*1.8

max_s_radius = **0.3**

# prepare data

# use the absolute values of all the indices

#sobol_indices = {key:np.abs(stats) for key, stats in sobol_indices.items()}

# dataframe with ST and S1

sobol_stats = {key:sobol_indices[key] **for** key **in** ['ST', 'S1']}

sobol_stats = pd.DataFrame(sobol_stats, index=problem['names'])

smax = sobol_stats.max().max()

smin = sobol_stats.min().min()

# dataframe with s2

s2 = pd.DataFrame(sobol_indices['S2'], index=problem['names'],

columns=problem['names'])

s2[s2<**0.0**]=**0.** #Set negative values to 0 (artifact from small sample sizes)

s2max = s2.max().max()

s2min = s2.min().min()

names = problem['names']

n = len(names)

ticklocs = np.linspace(**0**, **2***pi, n+**1**)

locs = ticklocs[**0**:-**1**]

filtered_names, filtered_locs = filter(sobol_indices, names, locs,

criterion, threshold)

# setup figure

fig = plt.figure()

ax = fig.add_subplot(**111**, polar=True)

ax.grid(False)

ax.spines['polar'].set_visible(False)

ax.set_xticks(ticklocs)

ax.set_xticklabels(names)

ax.set_yticklabels([])

ax.set_ylim(top=**1.4**)

legend(ax)

# plot ST

plot_circles(ax, filtered_locs, filtered_names, max_s_radius,

sobol_stats['ST'], smax, smin, 'w', 'k', **1**, **9**)

# plot S1

plot_circles(ax, filtered_locs, filtered_names, max_s_radius,

sobol_stats['S1'], smax, smin, 'k', 'k', **1**, **10**)

# plot S2

**for** name1, name2 **in** itertools.combinations(zip(filtered_names, filtered_locs), **2**):

name1, loc1 = name1

name2, loc2 = name2

weight = s2.loc[name1, name2]

lw = **0.5**+max_linewidth_s2*normalize(weight, s2min, s2max)

ax.plot([loc1, loc2], [**1**,**1**], c='darkgray', lw=lw, zorder=**1**)

**return** fig

**from** **matplotlib.legend_handler** **import** HandlerPatch

**class** **HandlerCircle**(HandlerPatch):

**def** **create_artists**(self, legend, orig_handle,

xdescent, ydescent, width, height, fontsize, trans):

center = **0.5** * width - **0.5** * xdescent, **0.5** * height - **0.5** * ydescent

p = plt.Circle(xy=center, radius=orig_handle.radius)

self.update_prop(p, orig_handle, legend)

p.set_transform(trans)

**return** [p]

**def** **legend**(ax):

some_identifiers = [plt.Circle((**0**,**0**), radius=**5**, color='k', fill=False, lw=**1**),

plt.Circle((**0**,**0**), radius=**5**, color='k', fill=True),

plt.Line2D([**0**,**0.5**], [**0**,**0.5**], lw=**8**, color='darkgray')]

ax.legend(some_identifiers, ['ST', 'S1', 'S2'],

loc=(**1**,**0.75**), borderaxespad=**0.1**, mode='expand',

handler_map={plt.Circle: HandlerCircle()})

sns.set_style('whitegrid')

fig = plot_sobol_indices(Si, criterion='ST', threshold=**0.005**)

fig.set_size_inches(**7**,**7**)

plt.show()

**Python script S3. Uncertainty Analysis**

**import** **random**

**import** **matplotlib.pyplot** **as** **plt**

**import** **pandas** **as** **pd**

**import** **statistics**

**import** **numpy** **as** **np**

**import** **scipy.stats** **as** **scs**

**from** **scipy.stats** **import** uniform

**from** **scipy.stats** **import** truncnorm

**import** **pandas** **as** **pd**

######### Generate input data phase ##################

**def** **get_truncated_normal**(low, upp):

mean = **0**

sd = **1**

**return** truncnorm(

(low - mean) / sd, (upp - mean) / sd, loc=mean, scale=sd)

**def** **get_uniform**(low, upp):

**return** np.linspace(uniform.ppf(low),

uniform.ppf(upp), **10000**)

inputX52 = []

**for** i **in** range(**10000**):

generator = get_truncated_normal(low=**0.65**, upp=**0.69**)

inputX52.append(generator.rvs())

inputX53 =[]

**for** i **in** range(**10000**):

generator = get_truncated_normal(low=**0.52**, upp=**0.57**)

inputX53.append(generator.rvs())

inputX16 =[]

**for** i **in** range(**10000**):

generator = get_truncated_normal(low=**0.2**, upp=**0.76**)

inputX16.append(generator.rvs())

inputX12 =[]

generator = get_uniform(low=**0.2**, upp=**0.6**)

**for** i **in** range(**10000**):

inputX12.append(generator[i])

parameters = []

**for** i **in** range(**10000**):

parameters.append([inputX52[i], inputX53[i], inputX16[i], inputX12[i]])

df1 = pd.DataFrame(parameters, columns=['yhanoconwwstream', 'yhairconwwstream',

'bhconwwstream', 'no2grohetconwwstream'])

**print**(df1)

writer = pd.ExcelWriter('UA.xlsx',

engine='xlsxwriter')

df1.to_excel(writer, sheet_name='UA')

writer.save()

########## GPS-X phase #############

current_row = **0**

simutation = **0**

# list to keep output values

BOD_outputs = []

TN_outputs = []

TSS_outputs = []

StopTime = **0.0**

# runs when new simulation started

**def** **start**():

**global** current_row, StopTime

**print**("Current row: {}".format(current_row))

sys.stdout.flush()

# set new variable values using dataframe row by index

# BOD_MoM_input10.iloc[current_row]['qadsconwwstream'] - means 'qadsconwwstream' cell of the row with index 'current_row'

gpsx.setTstop(StopTime)

gpsx.setValue('yhanoconwwstream', df1.iloc[current_row]['yhanoconwwstream'])

gpsx.setValue('yhairconwwstream', df1.iloc[current_row]['yhairconwwstream'])

gpsx.setValue('bhconwwstream', df1.iloc[current_row]['bhconwwstream'])

gpsx.setValue('no2grohetconwwstream', df1.iloc[current_row]['no2grohetconwwstream'])

# eor() function executed once at end of simulation

# finished set True is required to terminate the runSim() function

#

**def** **eor**():

# add bodweff final value to the list of BOD-outputs when simulation ended

**try**:

BOD_outputs.append(gpsx.getValue('bodwweff'))

TN_outputs.append(gpsx.getValue('tnwweff'))

TSS_outputs.append(gpsx.getValue('xwweff'))

**except** **Exception** **as** e:

# add info about exception to the result

BOD_outputs.append('Simulation failed: {}'.format(str(e)))

TN_outputs.append('Simulation failed: {}'.format(str(e)))

TSS_outputs.append('Simulation failed: {}'.format(str(e)))

**global** finished

finished = True

# cint() function executed at every communication interval

#

**def** **cint**():

# print each output value

**print**(gpsx.getValue('bodwweff'))

**print**(gpsx.getValue('tnwweff'))

**print**(gpsx.getValue('xwweff'))

sys.stdout.flush()

**pass**

**def** **start_iteration**():

gpsx.resetSim()

runSim()

# run simulation for each row in dataset

**for** i **in** range(len(df1)):

# get index of row and make it global

current_row = i

**try**:

simutation += **1**

**print**("Current simulation: {}/{}".format(str(simutation), str(len(df1))))

sys.stdout.flush()

# run simulatioion itself

# reset simultaion

**while** True:

**try**:

start_iteration()

**break**

**except** **Exception**:

**print**("Timeout reached! Trying again!")

sys.stdout.flush()

**continue**

**except** **Exception**:

**pass**

**print**(BOD_outputs)

**print**(TN_outputs)

**print**(TSS_outputs)

# create new column in dataframe i.e - 'Effluent BOD'

df1['Effluent BOD'] = BOD_outputs

df1['Effluent TN'] = TN_outputs

df1['Effluent TSS'] = TSS_outputs

writer = pd.ExcelWriter('Combined_uncertainty_outputs.xlsx',

engine='xlsxwriter')

sys.stdout.flush()

df1.to_excel(writer, sheet_name='Combined uncertainty outputs')

writer.save()

###### Results phase

df = pd.read_excel('Combined_uncertainty_outputs.xlsx', index_col=**0**)

**print**(df)

X1 = df['Effluent BOD']

X2 = df['Effluent TN']

X3 = df['Effluent TSS']

fig, (ax1, ax2, ax3) = plt.subplots(nrows=**3**, ncols=**1**, figsize=(**3**, **2**))

ax1.set_title('Effluent BOD', fontsize=**15**)

ax1.hist(X1.tolist(), **100**, facecolor='blue', alpha=**0.5**)

ax1.grid(axis='y', alpha=**0.75**)

ax1.set_xlabel('Effluent BOD (mg/L)', fontsize=**10**)

ax1.set_ylabel('Frequency', fontsize=**10**)

ax2.set_title('Effluent TN', fontsize=**15**)

ax2.hist(X2.tolist(), **100**, facecolor='blue', alpha=**0.5**)

ax2.grid(axis='y', alpha=**0.75**)

ax2.set_xlabel('Effluent TN (mg/L)', fontsize=**10**)

ax2.set_ylabel('Frequency', fontsize=**10**)

ax3.set_title('Effluent TSS', fontsize=**15**)

ax3.hist(X3.tolist(), **100**, facecolor='blue', alpha=**0.5**)

ax3.grid(axis='y', alpha=**0.75**)

ax3.set_xlabel('Effluent TSS (mg/L)', fontsize=**10**)

ax3.set_ylabel('Frequency', fontsize=**10**)

plt.subplots_adjust(bottom=**0.1**, right=**2.8**, top=**4.0**, hspace=**0.4**)

plt.show()

Mean_BOD1 = statistics.mean(X1)

Mean_BOD=round(Mean_BOD1, **2**)

STD_BOD1 = statistics.pstdev(X1, Mean_BOD)

STD_BOD=round(STD_BOD1, **2**)

RW_BOD1 = STD_BOD/Mean_BOD

RW_BOD=round(RW_BOD1, **2**)

CI_BOD1 = **1.96***STD_BOD

CI_BOD=round(CI_BOD1, **2**)

**print**("The combined standard uncertainty for effluent BOD is..", STD_BOD, "mg/L")

**print**("The expanded uncertainty for effluent BOD is..", CI_BOD, "mg/L")

**print**("The calculated effluent BOD is..", Mean_BOD, "±", CI_BOD, "mg/L (95% coverage interval)")

**print**("The relative width for effluent BOD is..", RW_BOD)

Mean_TN1 = statistics.mean(X2)

Mean_TN=round(Mean_TN1, **2**)

STD_TN1 = statistics.pstdev(X2, Mean_TN)

STD_TN=round(STD_TN1, **2**)

RW_TN1 = STD_TN/Mean_TN

RW_TN=round(RW_TN1, **2**)

CI_TN1 = **1.96***STD_TN

CI_TN=round(CI_TN1, **2**)

**print**("The combined standard uncertainty for effluent TN is..", STD_TN, "mg/L")

**print**("The expanded uncertainty for effluent TN is..", CI_TN, "mg/L")

**print**("The calculated effluent TN is..", Mean_TN, "±", CI_TN, "mg/L (95% coverage interval)")

**print**("The relative width for effluent TN is..", RW_TN)

Mean_TSS1 = statistics.mean(X3)

Mean_TSS=round(Mean_TSS1, **2**)

STD_TSS1 = statistics.pstdev(X3, Mean_TSS)

STD_TSS=round(STD_TSS1, **2**)

RW_TSS1 = STD_TSS/Mean_TSS

RW_TSS=round(RW_TSS1, **2**)

CI_TSS1 = **1.96***STD_TSS

CI_TSS=round(CI_TSS1, **2**)

**print**("The combined standard uncertainty for effluent TSS is..", STD_TSS, "mg/L")

**print**("The expanded uncertainty for effluent TSS is..", CI_TSS, "mg/L")

**print**("The calculated effluent TSS is..", Mean_TSS, "±", CI_TSS, "mg/L (95% coverage interval)")

**print**("The relative width for effluent TSS is..", RW_TSS)
